# Pulmonary hypertension in Finland 2008-2020: A descriptive real-world cohort study (FINPAH)

**DOI:** 10.1016/j.jhlto.2024.100191

**Published:** 2024-12-04

**Authors:** Markku Pentikäinen, Piia Simonen, Helena Tuunanen, Pauliina Leskelä, Terttu Harju, Pertti Jääskeläinen, Christian Asseburg, Minna Oksanen, Erkki Soini, Christina Wennerström, Airi Puhakka, on behalf of the FINPAH study group, Terttu Harju, Elina Heliövaara, Pertti Jääskeläinen, Katriina Kahlos, Pentti Korhonen, Tiina Kyllönen, Pauliina Leskelä, Kirsi Majamaa-Voltti, Piia Simonen, Anu Turpeinen, Helena Tuunanen, Ville Vepsäläinen, Tapani Vihinen

**Affiliations:** i Oulu University Hospital, Oulu, Finland; j Helsinki University Hospital and University of Helsinki, Helsinki, Finland; k Kuopio University Hospital, Kuopio, Finland; l Tampere University Hospital, Tampere, Finland; m Turku University Hospital, Turku, Finland; n Satasairaala Hospital, Pori, Finland; a Helsinki University Hospital and University of Helsinki, Helsinki, Finland; b Turku University Hospital, Turku, Finland; c Tampere University Hospital, Tampere, Finland; d Oulu University Hospital, Oulu, Finland; e Kuopio University Hospital, Kuopio, Finland; f ESiOR Oy, Kuopio, Finland; g Janssen-Cilag AB, Solna, Sweden; h Janssen-Cilag Oy, Espoo, Finland

**Keywords:** pulmonary arterial hypertension, chronic thromboembolic pulmonary hypertension, real-world evidence, registry study, survival

## Abstract

**Background:**

To assess characteristics, risk group distribution, and prognosis of patients with pulmonary arterial hypertension (PAH) or chronic thromboembolic pulmonary hypertension (CTEPH) in Finland.

**Methods:**

Clinical chart review of patients with PAH or CTEPH recorded between 2008 and 2019 and linkage to official mortality data.

**Results:**

We identified 627 patients, with 502 (80%) diagnosed after 2008, yielding an incidence of PAH and CTEPH of 4.0 and 2.9/million/year, respectively. The median time from symptoms to diagnosis was 1 year. Mean age at diagnosis of PAH patients (*n* = 268) was 57 years, 73% were women, 40% had idiopathic PAH, 28% associated with connective tissue diseases, and 15% with congenital heart disease, 9% had ≥3 cardiovascular comorbidities. At 1 year, 34%/34%/24%/8% were at the low/intermediate-low/intermediate-high/high Compera 2.0 risk classification groups. Survival was 91.3%, 74.8%, and 62.6% at 1, 3, and 5 years, respectively, with an improving trend over calendar time. Ten PAH patients had a lung transplant. PAH subtype, cardiac output, and the presence of ischemic heart disease or type 2 diabetes predicted survival.

CTEPH patients (*n* = 189) were 63 years (mean) at diagnosis and 49% were women. Of the CTEPH patients, 29% underwent pulmonary endarterectomy (PEA) and 22% were treated with balloon pulmonary angioplasty. Survival was 94.6%, 87.2%, and 79.4% at 1, 3, and 5 years, respectively. PEA patients were younger, had fewer comorbidities, and had longer survival than non-PEA patients.

**Conclusions:**

Incidence and survival of PAH and CTEPH patients in Finland were similar to previously presented data for other countries.

## Background

Pulmonary arterial hypertension (PAH) and chronic thromboembolic pulmonary hypertension (CTEPH) are clinically important and rare classes of pulmonary hypertension (PH). PAH is characterized by pulmonary vasoconstriction and remodeling and CTEPH by obstruction of pulmonary arteries with fibrotic material and vascular remodeling.^1^ The most common cause of death in PAH and CTEPH is right ventricular failure.^1^

Registries have been important in giving insight into the prevalence and incidence of PAH and CTEPH as well as the dismal prognosis of untreated PAH and CTEPH.^2^, ^3^ Predictors for survival have been identified from registries, and their combinations are now used to stratify patients and guide treatment.^1^, ^4^, ^5^ Importantly, registries have also revealed limited adherence to treatment guidelines, which urge the aggressive use of combination therapy for PAH and pulmonary endarterectomy (PEA) for CTEPH to improve patient prognosis.^1^

Nationwide real-world evidence of Finnish PAH and CTEPH patients is sparse and suggests very low incidence and prevalence of the diseases. A study from 2005 estimated a prevalence of 5.8 cases/million and an incidence of 0.2 to 1.3 cases/million/year for World Health Organization (WHO) group I PH.^6^ A nationwide, cross-sectional study survey^7^ identified 114 PAH patients and 51 CTEPH patients in the year 2010, with an estimated prevalence of 23.0/million. A quality-of-life study among PAH patients in Finland in 2012 identified 72 PAH and CTEPH patients in the largest PAH center, Helsinki University Hospital.^8^

Therefore, a real-world data study, FINPAH, was initiated to assess the current number of patients, their clinical characteristics, risk groups and patient prognosis, treatment pathways, and responses to treatment strategies in Finland. Here we present results on the number of patients, their clinical characteristics, risk groups, and prognosis.

## Materials and methods

### Study subjects

Patient-level data for the FINPAH cohort were collected by electronic chart review at all 5 Finnish university hospitals (Helsinki, Turku, Tampere, Oulu, and Kuopio). Because patients with suspected PH are referred to university hospitals, the FINPAH cohort is expected to cover the vast majority of Finnish PH patients. The study follows the Act on the Secondary Use of Health and Social Data (552/2019), with Finnish Social and Health Data Permit Authority (Findata) permit number THL/3614/14.02.00/2020. The study was retrospective and noninterventional, so patient consent was not required. Ethical approval was obtained from Helsinki University Hospital (HUS/2179/2020).

To be included in the FINPAH cohort, patients had to have a diagnosis of PAH (ICD-10 codes I27.0, I27.8, I27.9, Q21.8) or CTEPH (I27.2) or a prescription for prostacyclin analog (iloprost and treprostinil), prostacyclin receptor agonist (selexipag), an endothelin-receptor antagonist (ERA: bosentan, ambrisentan, sitaxentan, and macitentan), a phosphodiesterase 5 inhibitor (sildenafil and tadalafil) prescribed for PH, soluble guanylate cyclase stimulator (riociguat), or a calcium antagonist (calcium channel blocker: amlodipine, felodipine, nifedipine, and diltiazem) for treatment of PH, or symptoms obviously indicating PH (with later verification of PAH or CTEPH), recorded at least once in 2008-2019. All the patients included in the study did have a previous diagnosis of PAH or CTEPH. PAH and CTEPH diagnosis and subtypes were verified by expect clinicians using clinical, hemodynamic, laboratory, and imaging data according to 2015 European Society of Cardiology (ESC)/European Respiratory Society (ERS) Guidelines for the diagnosis and treatment of PH.^9^ Patients with prior lung transplant and data on patients younger than 18 years were excluded. For this publication, 2 study populations were defined: first PAH diagnosis in or after 2008 and first CTEPH diagnosis in or after 2008.

### Study design

Chart review was done by expert clinicians using electronic clinical research forms and spanned a wide range of variables, including demographics, comorbidities, clinical, hemodynamic, and laboratory data both at diagnosis and during follow-up, PAH medications and their dosing, hospitalizations, and interventions related to PH. Patient risks were assessed by calculating the ESC/ERS 2015 risk score^10^ and the Compera v2.0 risk scores.^11^ Completeness of the data at baseline for PAH and CTEPH were 95% and 96% for clinical parameters, 95% and 94% for right heart catheterization, 98% and 98% for echocardiography, and 71% and 68% for 6-minute walk test.

Clinical data were followed up until lung transplant, death, or the end of 2020, whichever happened first. Mortality data until the end of 2021 were obtained by linkage to the official Statistics Finland register. Mortality data were missing from 3 patients with PAH and 5 patients with CTEPH.

### Methods

Patient baseline characteristics, the distribution of risk scores at diagnosis and 1- and 2-years post diagnosis, and overall and hospitalization-free survival are described here. Risk scores were calculated using the last observation carried forward method, if there was at least 1 new observation of a relevant variable in a time window of ±1.5 months. Twelve patients whose survival data were unavailable were excluded from the survival analysis. Annual average incidence was estimated by dividing the observed number of incident cases in 2008-2019 by 5,525,292 (the size of the Finnish population at the end of 2019^12^) and by 12 for the number of years.

### Analysis

For continuous variables, means and standard deviations (SD) or 95% confidence intervals (95%CI) and medians and interquartile ranges (IQR) were reported, and counts and percentages for categorical variables. Estimated glomerular filtration rate was calculated using the Chronic Kidney Disease Epidemiology Collaboration (CKD-EPI) definition.^13^ Reference values for the 6-minute walking distance (6MWD) were calculated.^14^

Survival was defined as the time from first diagnosis to death, with the end of 2021 as a censoring time, and estimated using the Kaplan-Meier product limit estimator. Hospitalization-free survival was defined likewise for the event of death or hospitalization, whichever came first. The hospitalizations to perform PEA were not considered. Survival across risk groups was compared using the log-rank test, also for hospitalization-free survival. Wilcoxon signed-rank test was used to compare the numbers of comorbidities and age distributions of CTEPH patients undergoing PEA or not.

Characteristics potentially associated with survival were screened using univariable Cox proportional hazards regression analysis. The screening was carried out using both characteristics at diagnosis and 12-month follow-up, to improve the comprehensiveness of the data. At either time point, each characteristic was defined by the observation falling within a 3-month window and closest to the target time point. We included a variable to analysis of predictors only if 70% of patients had a recorded number of the variable for a continuous variable or recorded presence or absence for a dichotomous variable, to ensure representativeness of the study population.

Statistical analyses were done with R 4.0.3 in a secure operating environment. *p*-values below 0.05 were considered statistically significant. Due to the descriptive nature of this real-world evidence study, no calculations of statistical power were made, and *p*-values were not adjusted for multiple testing. To protect patient anonymity, results are not reported for N < 5 patients as required by Findata.^15^

## Results

The FINPAH cohort included a total of 627 patients. Of the 502 (80%) patients diagnosed in the year 2008 or later, the median duration of follow-up from diagnosis was 4.6 years for mortality (IQR 2.6-7.7, *n* = 494, 98.4%) data and 2.8 years for clinical and resource use (IQR 1.3-5.5, *n* = 502, 100.0%) data. The 2 study populations defined by PAH or CTEPH, respectively, diagnosed in 2008 or later, comprised 268 (53%) PAH patients and 189 (38%) CTEPH patients (Figure S1 in the Supplementary Appendix).

### Patient characteristics

The most frequent WHO group 1 PAH subtype was idiopathic PAH (40%), whereas 28% had PAH associated with connective tissue diseases and 15% had PAH associated with congenital heart disease (Table 1). The annual average incidence for PAH was approximately 4.0 and for CTEPH approximately 2.9 per million inhabitants, respectively.

**Table 1:** PH Classes and Subclasses for Patients Diagnosed in 2008 or Later

PH diagnosis	Number of patients (*n* = 502)
PAH, including the following subtypes	268 (53%)
IPAH	106 (21%, 40% of PAH)
HPAH	10 (2%, 4% of PAH)
APAH Connective tissue disease	76 (15%, 28% of PAH)
APAH Congenital heart disease	39 (8%, 15% of PAH)
APAH Portal hypertension	7 (1%, 3% of PAH)
APAH HIV^a^	<5 (<1%, <2% of PAH)
PVOD or PCH	12 (2%, 5% of PAH)
Drugs or toxins	7 (1%, 3% of PAH)
Other or unspecified^a^	<11 (2%, <4% of PAH)
CTEPH	189 (38%)
Other PH (associated with left heart disease, lung disease or hypoxia, unclear or multifactorial)	45 (9%)

Abbreviations: APAH, associated pulmonary arterial hypertension; CTEPH, chronic thromboembolic pulmonary hypertension; HIV, human immunodeficiency virus; HPAH, heritable pulmonary arterial hypertension; IPAH, idiopathic pulmonary arterial hypertension; PAH, pulmonary arterial hypertension; PCH, pulmonary capillary hemangiomatosis; PH, pulmonary hypertension; PVOD, pulmonary veno-occlusive disease.

a Specific numbers not given to protect anonymity.

Patient characteristics at diagnosis of PAH and CTEPH are shown in Table 2 and comorbidities in Table 3. As expected, the PAH cohort has a female predominance, and most patients had New York Heart Association (NYHA) III symptoms at diagnosis, despite a relatively short time from first symptoms to diagnosis. Vasoreactivity was found in 14.6% of the group I PAH patients.

**Table 2:** PAH and CTEPH Patient Characteristics at Diagnosis

Characteristic	PAH (*n* = 268^a^)	CTEPH (*n* = 189^a^)
Age, mean (SD)	57.4 (16.3)	63.0 (13.4)
Median (IQR)	60.6 (47.0-70.7)	66.1 (56.5-73.0)
Female sex, N (proportion)	195 (72.8%)	93 (49.2%)
BMI, kg/m^2^, mean (SD) Median (IQR)	27.5 (6.2) 26.7 (23.1-30.8)	28.8 (6.7) 27.3 (24.3-31.3)
Employment		
Full time	25.3%	28.6%
Retired	50.2%	57.8%
Other	24.5%	13.6%
Smoking		
Current	9.9%	9.5%
Stopped	33.2%	34.5%
Never	57.3%	56.5%
Time from first symptoms to diagnosis, years		
Mean (SD)	1.9 (3.6)	1.5 (1.7)
Median (IQR)	1.0 (0.6-2.1)	1.0 (0.6-1.8)
NYHA FC		
I	5.2%	0.0%
II	26.2%	33.3%
III	56.0%	55.0%
IV	12.5%	11.7%
Chest pain	15 (7%)	9 (6%)
Clinical right heart insufficiency	52 (22%)	27 (17%)
Systolic blood pressure, mm Hg, mean (SD)	130 (24)	131 (18)
Median (IQR)	126 (114-143)	130 (120-141)
Diastolic blood pressure, mm Hg, mean (SD)	79 (13)	81 (12)
Median (IQR)	79 (71-86)	80 (72-89)
Heart rate (1/min), mean (SD)	77 (14)	75 (14)
Median (IQR)	77 (69-85)	74 (65-81)
O_2_ saturation, %, mean (SD)	93.8 (4.7)	92.0 (11.2)
Median (IQR)	94 (92-97)	94 (91-96)
NT-proBNP, ng/liter, mean (SD)	2,389 (3,091)	1,916 (3,359)
Median (IQR)	1,493 (520-3124)	829 (255-2,043)
BNP, ng/liter, mean (SD)	428.1 (428.0)	212.4 (281.4)
Median (IQR)	292 (97-671)	98.5 (47.5-228)
Haemoglobin, mg/liter, mean (SD)	141.5 (21.0)	147.5 (18.2)
Median (IQR)	140 (127-154)	149 (138-158)
Creatinine, µmol/liter, mean (SD)	90.4 (71.8)	88.9 (26.1)
Median (IQR)	80.0 (66.5-96.0)	86.0 (74.0-98.3)
eGFR (CKD-EPI definition), ml/min/1.73 m^2^, mean		
(SD)	75.6 (24.4)	73.1 (19.5)
Median (IQR)	76.0 (58.0-92.0)	71.5 (60.4-100.5)
Alanine aminotransferase, U/liter, mean (SD)	38.3 (126)	39.7 (52.5)
Median (IQR)	25.5 (18.3-35.0)	32.0 (21.0-44.0)
EKG sinus rhythm	83.8%	93.8%
EKG RBBB	13.5%	10.5%
6-minute walking distance, m, mean (SD)	347.9 (151.6)	371.1 (143.4)
Median (IQR)	360 (240-461)	371 (263-477)
Percentage of predicted 6MWD	70.8 (28.4)	81.8 (28.1)
Right heart catheterization recorded, N (proportion)	255 (95.1%)	177 (93.7%)
mPAP (mm Hg), mean (SD)	46.7 (11.4)	44.6 (10.1)
Median (IQR)	47 (39-53)	45 (37-51)
dPAP (mm Hg), mean (SD)	29.5 (10.2)	25.8 (8.9)
Median (IQR)	29 (22-36)	24 (20-31)
PCWP (mm Hg), mean (SD)	10.8 (4.8)	9.9 (4.8)
Median (IQR)	10 (8-13)	9 (6-12)
CI (liter/min), mean (SD)	2.4 (0.8)	2.4 (0.9)
Median (IQR)	2.3 (1.9-2.8)	2.3 (1.8-2.8)
PVR (WU), mean (SD)	9.1 (4.3)	8.3 (4.0)
Median (IQR)	8.4 (6.0-12.0)	7.4 (5.5-10.2)
RA/CVP (mm Hg), mean (SD)	9.1 (10.1)	7.2 (4.0)
Median (IQR)	7.0 (5.0-11.8)	6.0 (4.0-10.0)
DLCOc %, mean (SD)	53.5 (19.3)	71.5 (16.6)
Median (IQR)	51 (39-67)	70 (63-82)
DLCOc <45%, N (proportion)	50 (36%)	<5 (<6%)
ECHO		
TAPSE, mm, mean (SD)	17.5 (5.0)	19.3 (5.0)
Median (IQR)	17.0 (14.0-20.5)	20.0 (16.5-22.5)
Pericardial fluid N, %	33 (18%)	10 (8%)
LV eccentric N, %	115 (78%)	76 (75%)
Caval respiratory variation N, %	98 (67%)	68 (71%)
Mutation status		
BMPR2	12 (4%)	<5 (<3%)
Other	<5 (<2%)	<5 (<3%)
None	17 (6%)	6 (3%)
Not tested^b^	>235 (>87%)	>175 (>92%)

Abbreviations: 6MWD, 6-minute walking distance; BMI, body mass index; BMPR2, bone morphogenetic protein receptor type II; BNP, brain natriuretic peptide; CI, cardiac index; CTEPH, chronic thromboembolic pulmonary hypertension; DLCOc, diffusing capacity of the lungs for carbon monoxide, adjusted for hemoglobin; dPAP, diastolic pulmonary arterial pressure; ECHO, echocardiography; eGFR, estimated glomerular filtration rate; EKG, electrocardiogram; IQR, interquartile ranges; LV, left ventricle; mPAP, mean pulmonary arterial pressure; NT-proBNP, N-terminal prohormone of BNP; NYHA FC, New York Heart Association Functional Class; PAH, pulmonary arterial hypertension; PCWP, pulmonary capillary wedge pressure; PVR, pulmonary vascular resistance; RA/CVP, right atrial/central venous pressure; RBBB, right bundle-branch block; SD, standard deviation, TAPSE, tricuspid annular plane systolic excursion.

a Data for some patients were missing for employment, smoking status, and clinical examinations.

b Exact numbers not reported to protect anonymity.

**Table 3:** Comorbidities of PAH and CTEPH Patients at Diagnosis

Diagnosis, N (proportion)	PAH (*n* = 268)	CTEPH (*n* = 189)
Major cardiovascular comorbidities		
Treated hypertension	101 (38%)	95 (50%)
Diabetes	52 (19%)	26 (14%)
Stroke	7 (3%)	9 (5%)
Obesity (BMI ≥30)	49 (18%)	45 (24%)
Ischemic heart disease	34 (13%)	22 (12%)
3 or more major cardiovascular comorbidities^a^	23 (9%)	15 (8%)
Cardiopulmonary phenotype (DLCOc <45% and smoking history)	25 (9%)	<5 (<3%)
Atrial fibrillation	47 (18%)	17 (9%)
Atrial flutter	9 (3%)	<5 (<3%)
Chronic kidney disease	14 (5%)	6 (3%)
Hematological disease	12 (4%)	17 (9%)
Innate heart defect	40 (15%)	<5 (<3%)
ASD	17 (6%)	<5 (<3%)
AVSD	<5 (<2%)	0
VSD	5 (2%)	<5 (<3%)
PDA	<5 (<2%)	0
Fontan or UVH	<5 (<2%)	0
Eisenmenger	5 (2%)	0
Corrected heart defect	10 (4%)	0
Lung disease	78 (29%)	54 (29%)
Asthma	34 (13%)	38 (20%)
Parenchymal	17 (6%)	<5 (<3%)
COPD or emphysema	13 (5%)	17 (9%)
Other	26 (10%)	8 (4%)
Rheuma	90 (34%)	10 (5%)
Scleroderma or CREST	54 (20%)	0
Sjögren	13 (5%)	<5 (<3%)
Rheumatoid arthritis	6 (2%)	7 (4%)
Other (including MCTD)	22 (8%)	<5 (<3%)
Sleep apnoea	23 (9%)	17 (9%)
Valvular disease (moderate or severe)	36 (13%)	14 (7%)
Tricuspid regurgitation	24 (9%)	8 (4%)
Other	12 (4%)	6 (3%)
Any CTEPH risk factor	102 (38%)	179 (95%)
Cancer	24 (9%)	28 (15%)
Deep vein thrombosis	16 (6%)	57 (30%)
Hypothyroidism	43 (16%)	19 (10%)
History of lung embolism	31 (12%)	174 (92%)
Thrombophilia	<5 (<2%)	17 (9%)
TOS	0	<5 (<3%)
Pacemaker	6 (2%)	7 (4%)
VP shunt	0	<5 (<3%)

Abbreviations: ASD, atrial septal defect; AVSD, atrioventricular septal defect; BMI, body mass index; COPD, chronic obstructive pulmonary disease; CREST, limited cutaneous form of systemic sclerosis; CTEPH, chronic thromboembolic pulmonary hypertension; DLCOc, diffusing capacity of the lungs for carbon monoxide, adjusted for hemoglobin; MCTD, mixed cutaneous tissue disease; PAH, pulmonary arterial hypertension; PDA, patent ductus arteriosus; UVH, univentricular heart; VP, ventriculoperitoneal; VSD, ventricular septal defect; TI, tricuspid insufficiency; TOS, thoracic outlet syndrome.

a Three or more of the following: treated hypertension, diabetes, stroke, obesity (BMI ≥ 30), ischemic heart disease.

Notably, 92% of the CTEPH patients had a history of pulmonary embolism, and many had risk factors for thrombosis. Genetic testing results were available for <45 patients (<10%), and a mutation in the bone morphogenetic protein receptor type II gene was present in 12 (5%, about 40% of tested) of PAH patients and <5 of CTEPH patients (tested because of relatives with known mutations, for example). Other known mutations were present in fewer than 5 patients.

In the CTEPH patients, 55 PEAs (29%) were recorded during follow-up, and 41 patients (22%) underwent balloon pulmonary angioplasty (BPA). After PEA, 7 patients (13% of PEA-treated patients) received BPA for residual PH. No patients in the WHO group I underwent BPA or PEA.

Survival was higher in patients undergoing PEA (*p* = 0.001, Figure 3). PEA patients were younger than non-PEA patients (*p* < 0.001) and had fewer comorbidities (*p* = 0.02). Non-PEA patients were treated with BPA (26%) or drugs (80%). Lung transplants were recorded in 10 (3.7%) PAH patients and <5 (<2.2%) CTEPH patients. Data are not presented here in detail.

### Prognosis

**Figure 1:**
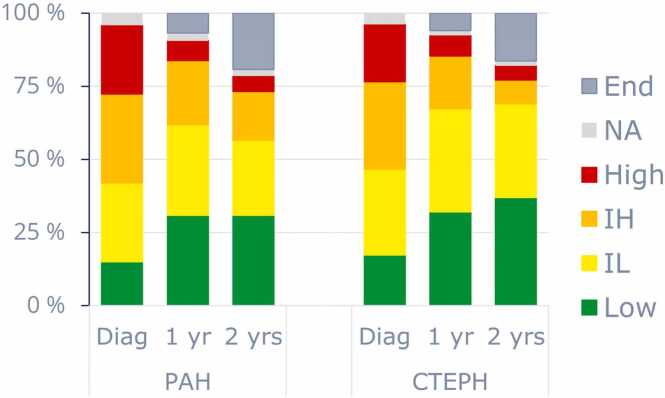
Compera v2.0 4-strata risk score at diagnosis, 1, and 2 years. The 4 strata are low, intermediate-low (IL), intermediate-high (IH), and high risk.^4^ NA indicates alive with missing values and End represents patients no longer in follow-up (i.e., dead or censored). CTEPH, chronic thromboembolic pulmonary hypertension; PAH, pulmonary arterial hypertension.

ESC/ERS 2015 risk scores could be determined at diagnosis for 99% of patients and Compera v2.0 4-strata risk scores for 96% of patients. The distributions of ESC/ERS 2015 risk scores at diagnosis were 27%/64%/9% of PAH patients at low/intermediate/high risk and 34%/61%/5% of CTEPH patients, respectively. The distributions of Compera v2.0 risk scores are shown in Figure 1. The proportion of patients at low risk, which is the treatment goal, significantly increased and similarly the proportion of patients at high risk decreased from diagnosis to 2 years, both for PAH (16%/28%/32%/24% at diagnosis to 39%/33%/21%/7% at 2 years) and CTEPH (18%/31%/31%/20% at diagnosis to 45%/39%/10%/6% at 2 years). Similar risk reductions were evident using ESC/ERS 2015 and Compera 3-strata models (not shown).

**Figure 2:**
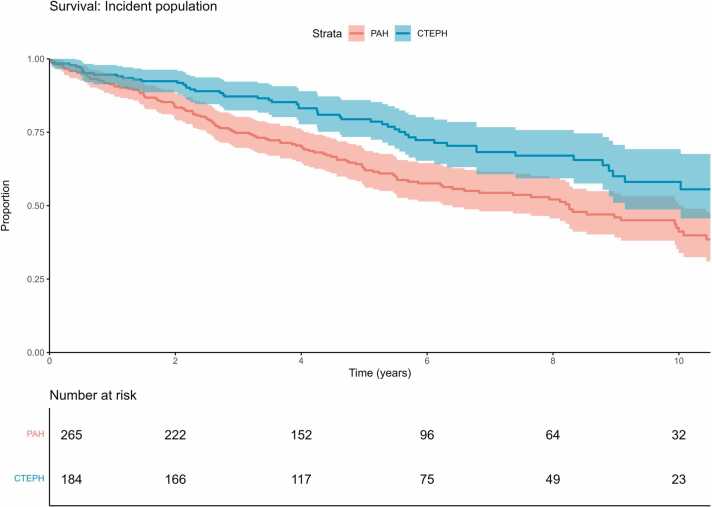
Estimated overall survival in PAH and CTEPH patients. CTEPH, chronic thromboembolic pulmonary hypertension; PAH, pulmonary arterial hypertension.

Survival could be assessed for 265 PAH patients and 184 CTEPH patients (99% and 97% of the patients, respectively), with the Kaplan-Meier estimates shown in Figure 2. Survival in PAH patients was 91.3% (95%CI 88.0%-94.8%) at 1 year, 74.8% (95%CI 69.7%-80.2%) at 3 years, and 62.6% (95%CI 56.7%-69.0%) at 5 years. Survival in CTEPH patients was 94.6% (CI 91.3%-97.9%) at 1 year, 87.2 (95%CI 82.4%-92.2%) at 3 years, and 79.4% (95%CI 73.4%-85.9%) at 5 years.

No significant improvement in survival with calendar time was observed, but a trend was noted (Figures S2 and S3 in the Supplementary Appendix). Thus, in PAH patients diagnosed before vs after September 30, 2015 (when the 2015 ESC/ERS PH Guidelines with recommendations for combination therapy), survival improved from 90.1% to 93.3% at 1 year, from 71.6% to 79.9% at 3 years, and from 61.1% to 61.9% at 5 years (*p* = 0.26), and use of the ERA+phosphodiesterase 5 inhibitor treatment combination as first PH therapy increased from 5.0% to 29.4%. In CTEPH patients, survival improved from 93.3% to 96.2% at 1 year, from 84.8% to 90.3% at 3 years, and from 77.1% to 80.9% at 5 years (*p* = 0.36). Riociguat was used more frequently as the first medical therapy for CTEPH (from 10.1%-42.9%), and other changes in treatment patterns may also explain these observations.

Survival differed by ESC/ERS 2015 risk assessment groups at diagnosis, both in PAH (*p* < 0.001) and CTEPH (*p* < 0.001), with higher risk correlating with shorter survival. Kaplan-Meier estimates are shown in Figure 4. For example, 1-year survival was estimated at 98.6% (95%CI 95.8%0100.0%), 90.5% (86.2%-95.1%), and 72.7% (56.3%-93.9%) in PAH patients with low, intermediate, and high risk, respectively, and at 98.4% (95.2%-100.0%), 92.9% (88.3%-97.8%), and 88.9% (70.6%-100.0%) in CTEPH patients with low, intermediate, and high risk, respectively.

**Figure 3:**
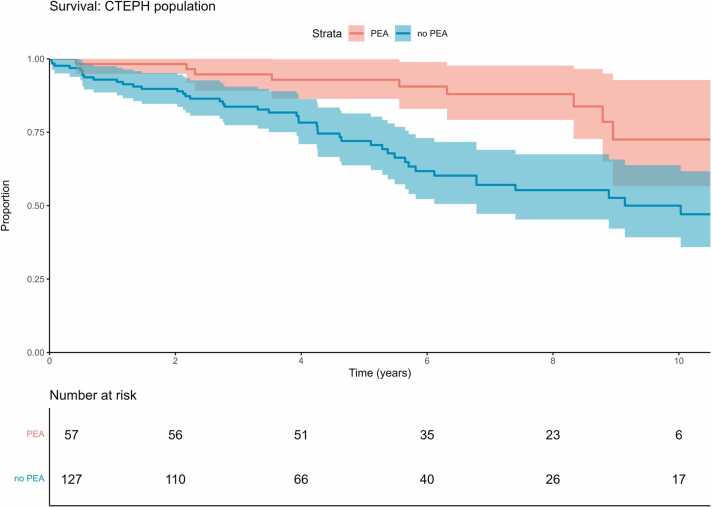
Estimated overall survival by PEA in CTEPH patients. CTEPH, chronic thromboembolic pulmonary hypertension; PEA, pulmonary endarterectomy.

**Figure 4:**
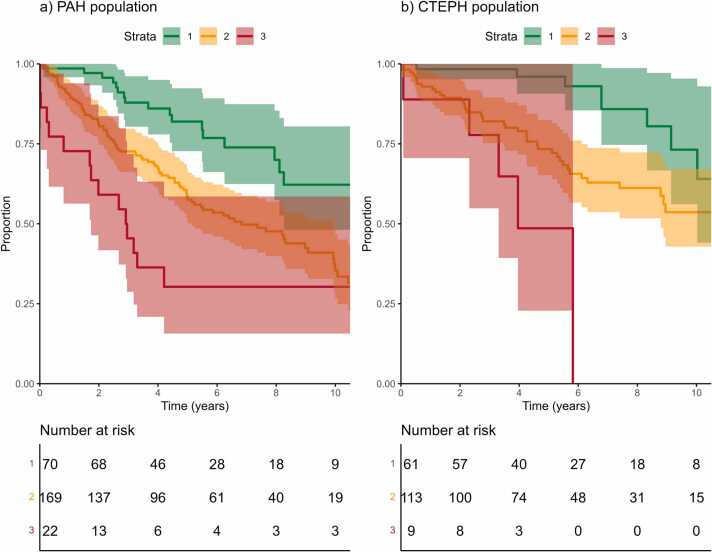
Estimated overall survival by ESC/ERS 2015 risk in (a) PAH and (b) CTEPH patients. CTEPH, chronic thromboembolic pulmonary hypertension; ESC/ERS, European Society of Cardiology/European Respiratory Society; PAH, pulmonary arterial hypertension.

Hospitalization-free survival (Figure S4 in the Supplementary Appendix) in PAH patients was 72.8% (95%CI 67.7%-78.4%) at 1 year and 41.2% (95%CI 35.4%-48.1%) at 5 years. Hospitalization-free survival in CTEPH patients was 74.5% (95%CI 68.4%-81.0%) at 1 year and 54.5% (95%CI 47.6%-62.5%) at 5 years. As expected, hospitalization-free survival decreased with increasing ESC/ERC 2015 risk both for PAH and CTEPH (data not shown).

### Predictors

Factors associated with survival were assessed both for PAH and CTEPH (Supplementary Table S1). We found associations with known demographic risk groups (age at diagnosis, date of diagnosis, scleroderma/CREST), comorbidities (hypertension, type 2 diabetes, ischemic heart disease, transient ischemic attack/stroke, kidney failure, low diffusing capacity of the lungs for carbon monoxide [DLCO] or cardiopulmonary phenotype, atrial arrhythmias), modifiable risk factors included in the ESC risk stratification (functional class, natriuretic peptides, 6MWD, right heart failure, hemodynamics, pericardial effusion), factors in REVEAL 2.0 (kidney function, functional class, blood pressure, 6MWD, natriuretic peptides, right atrial pressure [caval respiratory variation] and pulmonary vascular resistance). In addition, patients with significant tricuspid regurgitation, low tricuspid annular plane systolic excursion (TAPSE), right bundle-branch block (RBBB), and eccentric left ventricle in ECHO appeared to have a worse prognosis. In CTEPH, presentation with chest pain, cancer, and the presence of congenital heart defect were risk factors and, not unexpectedly, patients who underwent PEA surgery had a better prognosis.

## Discussion

This is the first systematic and comprehensive description of the PAH and CTEPH patient populations in Finland. Extensive data were collected in an electronic chart review, and the number of patients, their clinical characteristics, risk groups, and prognosis were analyzed.

The study suggested that the incidences of PAH (4/million) and CTEPH (3/million) in Finland are at the lower end of the range reported in other western countries,^16^ but close to that of the SPAHR registry in Sweden,^17^ which reported incidences of PAH 5 to 7 and CTEPH 2 to 4/million. Although PAH and CTEPH patients in our study were systematically identified at university hospitals, it is possible that some patients are treated and followed in smaller hospitals despite national and international guidance.

The diagnostic delays of PAH and CTEPH are notoriously long.^1^ Here, diagnosis of PAH and CTEPH was reached 1 year (median) after the onset of symptoms. This is at the shorter end of the spectrum; a diagnostic delay of up to 44 months was reported previously in Australia.^18^ The fact that over 30% of our patients had NYHA I or II symptoms at diagnosis is also suggestive of a rather early diagnosis. However, more than 50% were in FC III and almost 13% in FC IV, that is, in high risk. The age at diagnosis of PAH patients has increased over time, and means 57 years (PAH) and 63 years (CTEPH) is in line with that of other contemporary registries but still younger than in SPAHR,^17^ which reported ca. 67 years (PAH) and 71 years (CTEPH).

Comorbidities are of special importance as they affect treatment response and prognosis.^4^, ^19^, ^20^ The type and number of cardiovascular comorbidities in PAH patients were not unexpected. Around 20% of PAH patients had atrial fibrillation or flutter and 9% had 3 or more major cardiovascular comorbidities, suggesting a “left-heart phenotype.” Type 2 diabetes, ischemic heart disease, and a history of stroke were negatively associated with patient survival, consistent with previous findings.^19^, ^21^ In addition, 9% of PAH patients had a “cardiopulmonary phenotype,” which was previously identified as a high-risk subgroup.^11^ In this cohort, comorbidities affected prognosis (Table S1) but did not affect initial treatment strategy (mono vs dual therapy) (not shown).

Comorbidities in CTEPH patients are also important; the patients are older than PAH patients and an assessment of comorbidities determines whether they are considered candidates for PEA surgery. Surprisingly, the profile of comorbidities of CTEPH patients was quite similar to that of PAH patients. Expectedly, cancer, a prothrombogenic condition, was more prevalent in CTEPH and an expected marker of poor prognosis. Of the comorbidities, chronic kidney disease was also a marker for poor prognosis in CTEPH, consistent with a previous report.^22^

Risk stratification has become an important part of patient care, both for the analysis of patient prognosis and for guiding treatment. Although risk stratification was not documented in the patient records, the good availability of variables required for the ESC/ERS and Compera risk scores allowed risk stratification both at the time of diagnosis and at follow-up. At the time of diagnosis, ESC/ERS 2015 risk class predicted mortality well, both for PAH and CTEPH. The distribution of risk classes at diagnosis of PAH patients was close to that found in the COMPERA registry,^4^ and at follow-up even more patients reached low (34% FINPAH vs 17% COMPERA) and intermediate-low (34% FINPAH vs 28% COMPERA) classes. Survival of PAH patients was similar to that in COMPERA and close to that seen by the Pulmonary Hypertension Society of Australia and New Zealand,^18^ especially when focusing on patients diagnosed after 2015.

Survival of CTEPH cohorts depends much on the proportion of patients undergoing PEA surgery. In this cohort, 29% of CTEPH patients underwent PEA, which is the same as was reported for Sweden (30% in SPAHR). However, 1, 3, and 5-year survival seemed to be slightly better in our study (FINPAH PEA 98.2%, 94.7%, 92.9%, non-PEA 92.9%, 83.7%, 72.0%) compared to SPAHR (PEA 94%, 90%, 90%, non-PEA 94%, 78%, 66%).^17^

The analysis of predictors for survival can be of value in improving risk stratification, especially if the predictors are modifiable. Our results suggest that blood pressure and kidney function, which are included in REVEAL 2.0 and REVEAL Lite 2, and DLCO and pulmonary vascular resistance, which are included in REVEAL 2.0, could potentially be valuable additions to ESC/COMPERA, but their independent incremental value must be studied. In addition, this study highlights the known values of electrocardiogram^23^ (atrial arrhythmias, RBBB) and ECHO^24^ (TAPSE and LV eccentricity) in providing additional prognostic information. Short hospitalization-free survival indicates a high burden for specialized health care, as approximately 30% of the PAH patients are hospitalized in 1 year. However, hospitalization in this study at 1 year was lower than in the Opus/Orpheus registry, where 60% of the patients were free from hospitalization in 1 year.^25^ Similar to overall survival, hospitalization-free survival strongly depended on the patient's ESC/COMPERA risk class. Interestingly, hospitalization-free survival was similar in PAH and CTEPH patients, although CTEPH patients undergoing PEA fared significantly better than non-PEA patients (data not shown).

The strength of this study is that it evaluated a wide range of clinical, laboratory, and imaging parameters that allow future analysis of potential predictive factors. The weakness of this study is that not all variables were available systematically in real-world data. Although data on race were not collected, the vast majority of the study population identifies as White race and this limits the generalizability of the results to other populations.

## Conclusions

The Finnish PAH and CTEPH cohorts were larger than expected, when compared to the sparse reports from previous years. There were fewer hospitalizations compared to other registries. Survival was in line with other contemporary cohorts (e.g., SPAHR), and it seemed to improve over time.

## Author contributions

Markku Pentikäinen: Conceptualization, Methodology, Resources, Investigation, Writing—original draft. Piia Simonen: Conceptualization, Resources, Investigation, Writing—review and editing. Helena Tuunanen: Resources, Investigation. Pauliina Leskelä: Resources, Investigation, Writing—review and editing. Terttu Harju: Resources, Investigation, Writing—review and editing. Pertti Jääskeläinen: Resources, Investigation, Writing—review and editing. Christian Asseburg: Formal analysis, Methodology, Writing—original draft. Minna Oksanen: Formal analysis, Writing—original draft, Visualization. Erkki Soini: Project administration, Methodology, Formal analysis, Writing—review and editing. Christina Wennerström: Methodology, Validation, Writing—review and editing. Airi Puhakka: Conceptualization, Funding acquisition, Project administration, Writing—original draft.

## Disclosure statement

The authors declare the following financial interests/personal relationships which may be considered as potential competing interests: Markku Pentikainen reports financial support was provided by State Research Funding (VTR) granted to Helsinki University Hospital. Markku Pentikainen reports financial support was provided by Janssen-Cilag OY. Piia Simonen reports financial support was provided by State Research Funding (VTR) granted to Helsinki University Hospital. Piia Simonen reports financial support was provided by Janssen-Cilag OY. Pauliina Leskela reports financial support was provided by Janssen-Cilag OY. Markku Pentikainen reports a relationship with Orion Corporation that includes equity or stocks. Markku Pentikainen reports a relationship with Janssen-Cilag OY that includes consulting or advisory, speaking and lecture fees, and travel reimbursement. Markku Pentikainen reports a relationship with MSD that includes consulting or advisory. Pauliina Leskela reports a relationship with Janssen-Cilag OY that includes speaking and lecture fees and travel reimbursement. Pauliina Leskela reports a relationship with Abbott that includes travel reimbursement. Pauliina Leskela reports a relationship with Medtronic that includes travel reimbursement. Terttu Harju reports a relationship with Janssen-Cilag OY that includes speaking and lecture fees and travel reimbursement. Terttu Harju reports a relationship with Nordic Infucare that includes travel reimbursement. Pertti Jaaskelainen reports a relationship with Janssen-Cilag OY that includes speaking and lecture fees and travel reimbursement. Airi Puhakka reports a relationship with Johnson & Johnson that includes equity or stocks. Airi Puhakka reports a relationship with Idorsia that includes equity or stocks. Airi Puhakka reports a relationship with Bittium that includes equity or stocks. The other authors declare that they have no known competing financial interests or personal relationships that could have appeared to influence the work reported in this paper.

We acknowledge Dr Sandra Hänninen, ESiOR Oy, for language proofreading. We acknowledge all participating patients and clinical staff. We acknowledge M. Kukkonen from HUS, L.-P. Lyytikäinen from TAYS, H. Lähdeaho from TAYS, P. Mankinen from ESiOR Oy, T. Vasankari from TYKS, H. Vihinen from TYKS, and M. Virtanen from TAYS for their contribution to data collection.

This study was funded by Janssen-Cilag Oy, Espoo, Finland. M.P. and P.S. were funded by State Research Funding (VTR) granted to Helsinki University Hospital.

## Footnotes

Appendix A Supplementary data associated with this article can be found in the online version at doi:10.1016/j.jhlto.2024.100191.
