# Supplementary material for: Pulmonary hypertension in Finland 2008-2020: A descriptive real-world cohort study (FINPAH)
Source: JHLT Open. 2024 Dec 4;7:100191. doi: 10.1016/j.jhlto.2024.100191 (PMC11935358; doi:10.1016/j.jhlto.2024.100191)
Supplement: Supplementary file 1 — Supplementary material [file mmc1.docx]

# Supplement to Pentikäinen M, et al. Pulmonary hypertension in Finland 2008-2020: a descriptive real-world cohort study (FINPAH)


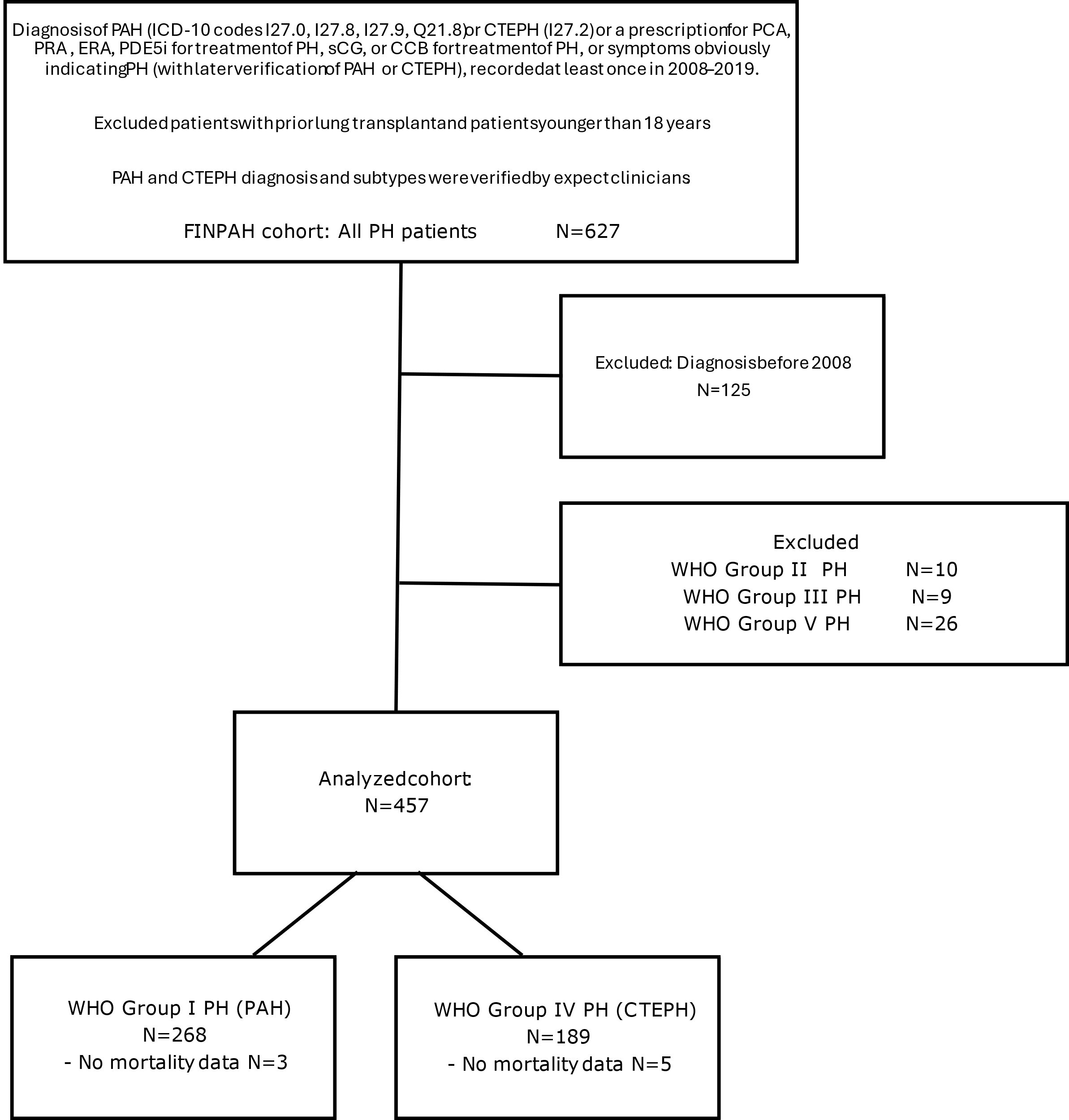


**Figure S1. Patient flowchart**


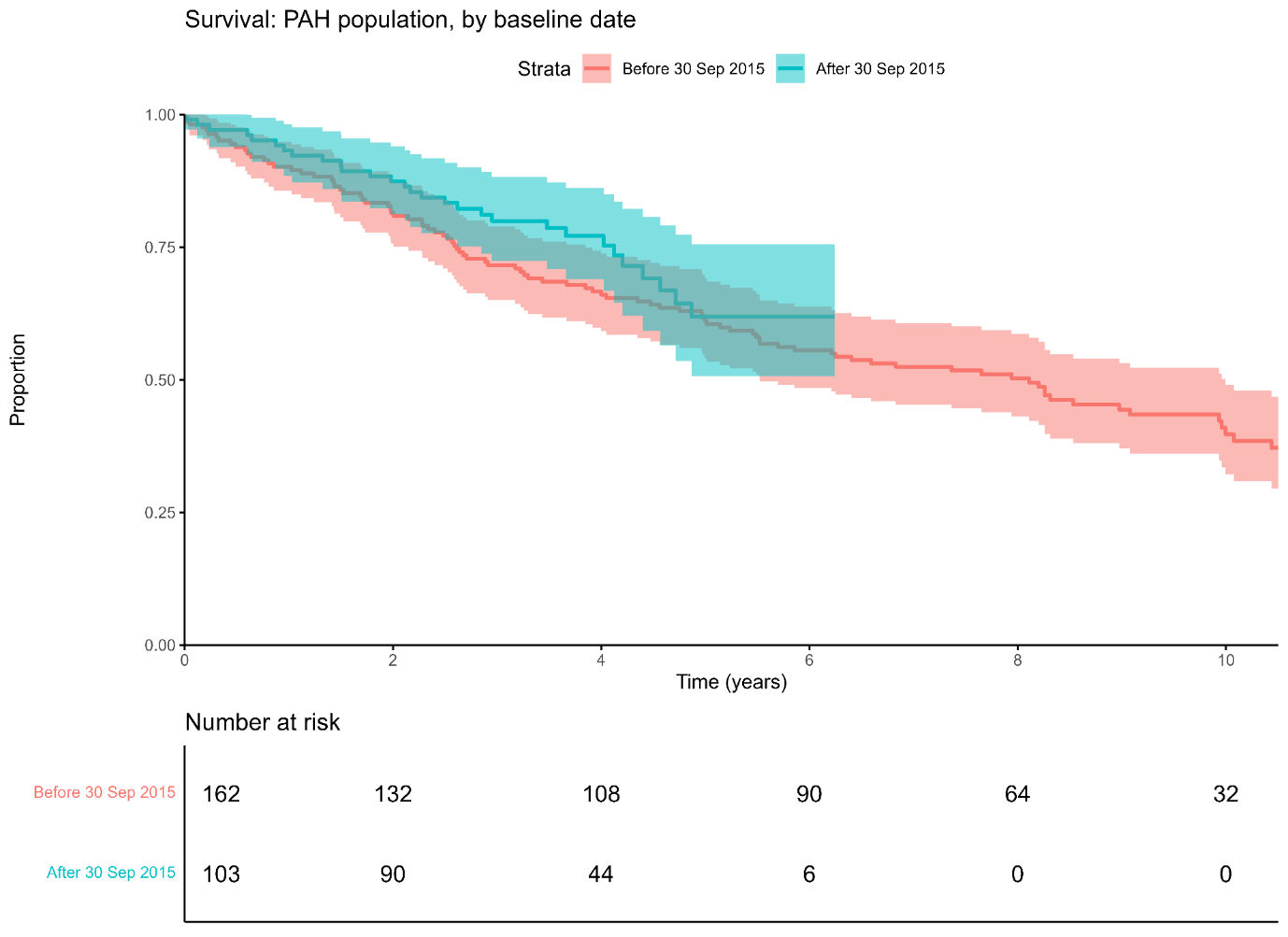


**Figure S2. Overall survival in PAH patients, by baseline date**


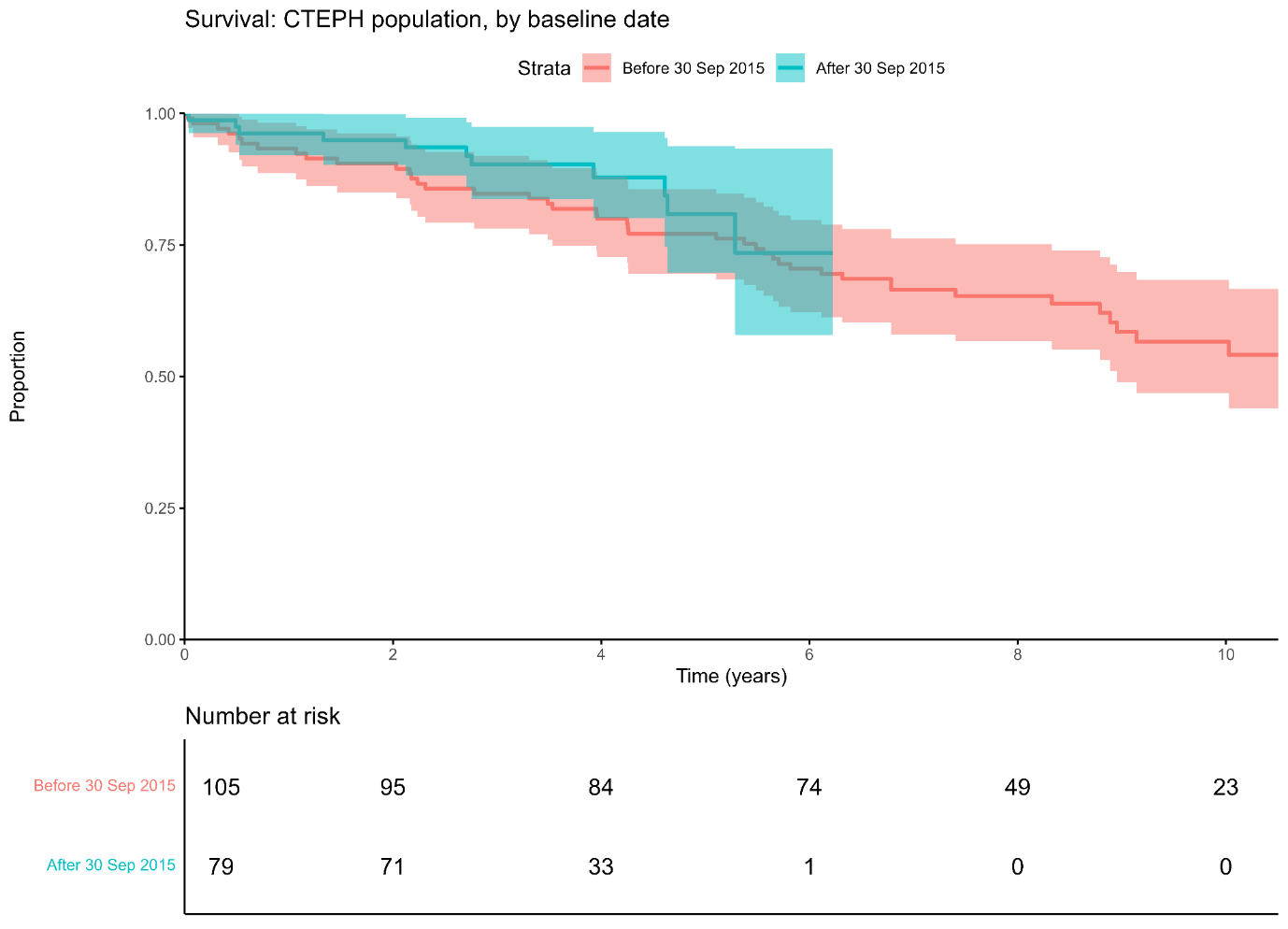


**Figure S3. Overall survival in CTEPH patients, by baseline date**


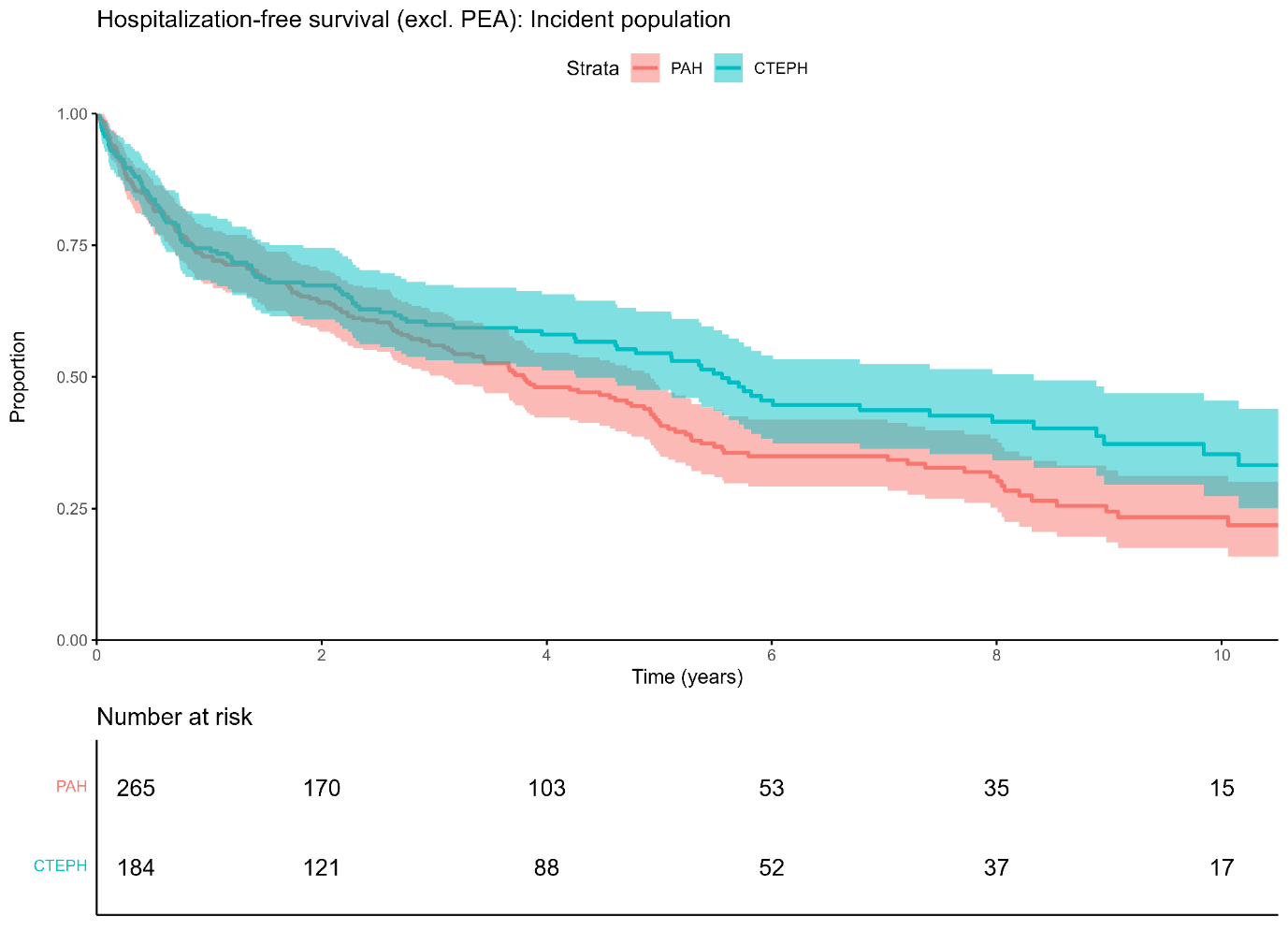


**Figure S4. Hospitalization-free survival in PAH and CTEPH patients, excluding hospitalization related to pulmonary endarterectomy (PEA)**

**Table S1**. Characteristics associated with overall survival at diagnosis and at 12 months using univariable analysis with a p-value less than 0.05 and coverage ≥70%. Other tested characteristics are listed in the supplementary appendix.

| Characteristics | Cox PH summary at diagnosis (Hazard ratio, 95% CI) | Cox PH summary at 12 months (Hazard ratio, 95% CI) |
| --- | --- | --- |
| PAH  Number of patients at diagnosis=265, at 12 months=242. Total number of deaths=126 | | |
| Age at diagnosis | 1.05 (1.03 – 1.06), p<0.001 | 1.05 (1.03 – 1.06), p<0.001 |
| Current smoking or snuff | 0.58 (0.27 – 1.26), p=0.17 | 0.29 (0.09 – 0.92), p=0.04 |
| NYHA FC 1 or 2 (reference) | 1.00 | NA |
| NYHA FC 3 | 1.60 (1.02 – 2.51), p=0.04 | NA |
| NYHA FC 4 | 2.05 (1.12 – 3.75), p=0.02 | NA |
| Clinical right heart failure | 1.80 (1.19 – 2.73), p=0.006 | 2.44 (1.51 – 3.95), p<0.001 |
| NT-proBNP (ng/l) /1000* | 1.09 (1.04 – 1.14), p<0.001 | NA |
| eGFR | 0.98 (0.98 – 0.99), p<0.001 | NA |
| ECG: Atrial fibrillation | 1.43 (0.84 – 2.44), p=0.19 | 2.45 (1.49 – 4.03), p<0.001 |
| Right bundle branch block | 1.34 (0.78 – 2.30), p=0.29 | 2.14 (1.23 – 3.74), p=0.008 |
| 6MWD m/100* | 0.72 (0.62 – 0.83), p<0.001 | NA |
| Cardiac output | 0.85 (0.73 – 0.99), p=0.04 | NA |
| Caval respiratory variation | NA | 0.37 (0.23 – 0.62), p<0.001 |
| DLCOc <45% | 2.09 (1.40 – 3.14), p<0.001 | 0.94 (0.30 – 2.98), p=0.98 |
| Treated hypertension | 1.40 (0.98 – 2.00), p=0.06 | 1.56 (1.05 – 2.30), p=0.03 |
| Type 2 diabetes | 2.08 (1.38 – 3.14), p<0.001 | 2.22 (1.40 – 3.51), p<0.001 |
| Stroke | 2.35 (0.95 – 5.78), p=0.06 | 3.37 (1.36 – 8.37), p=0.009 |
| Ischemic heart disease | 2.09 (1.33 – 3.26), p=0.001 | 2.43 (1.50 – 3.94), p<0.001 |
| Cardiopulmonary phenotype | 1.99 (1.18 – 3.35), p=0.009 | 0.75 (0.10 – 5.43), p=0.78 |
| Scleroderma or CREST | 3.30 (2.21 – 4.92), p<0.001 | 3.46 (2.19 – 5.45), p<0.001 |
| Tricuspid insufficiency (moderate or severe) | 1.76 (1.02 – 3.02), p=0.04 | 1.82 (0.99 – 3.32), p=0.05 |
| Transient ischemic attack | 2.59 (0.95 – 7.08), p=0.06 | 4.02 (1.46 – 11.08), p=0.007 |
|  |  |  |
| CTEPH  Number of patients at diagnosis=184, at 12 months=174. Total number of deaths=55 | | |
| First diagnosis date (years) | 0.90 (0.81 – 0.99), p=0.03 | 0.92 (0.82 – 1.04), p=0.18 |
| Age at diagnosis | 1.08 (1.04 – 1.11), p<0.001 | 1.10 (1.05 – 1.14), p<0.001 |
| NYHA FC 1 or 2 (reference) | 1.00 | NA |
| NYHA FC 3 | 1.71 (0.78 – 3.77), p=0.18 | NA |
| NYHA FC 4 | 6.83 (2.82 – 16.54), p<0.001 | NA |
| Chest pain | 2.11 (0.75 – 5.94), p=0.16 | 3.02 (1.06 – 8.60), p=0.04 |
| Clinical right heart insufficiency | 3.60 (1.84 – 7.04) p<0.001 | 3.27 (1.55 – 6.90), p=0.002 |
| Diastolic blood pressure | 0.97 (0.94 – 0.99), p=0.01 | NA |
| NT-proBNP (ng/l) /1000* | 1.14 (1.09 – 1.20), p<0.001 | NA |
| Hemoglobin | 0.97 (0.95 – 0.98), p<0.001 | NA |
| Creatinine | 1.01 (1.00 – 1.02), p=0.003 | NA |
| eGFR | 0.97 (0.95 – 0.99), p<0.001 | NA |
| Alanine aminotransferase | 1.00 (1.00 – 1.01), p=0.008 | NA |
| ECG: Sinus rhythm | 0.31 (0.13 – 0.74), p=0.008 | 0.42 (0.18 – 1.00), p=0.05 |
| ECG: Atrial fibrillation | 4.69 (1.62 – 13.55), p=0.004 | 4.55 (1.58 – 13.10), p=0.005 |
| ECG: Atrial flutter | 8.72 (1.16 – 65.51), p=0.04 | 1.11 (0.15 – 8.10), p=0.92 |
| PAP mean | 1.03 (1.00 – 1.06), p=0.03 | NA |
| PAP diastolic | 1.04 (1.01 – 1.07), p=0.006 | NA |
| PCWP | 1.10 (1.06 – 1.15), p<0.001 | NA |
| PVR | 1.07 (1.00 – 1.14), p=0.04 | NA |
| TAPSE | 0.90 (0.84 – 0.97), p=0.004 | NA |
| Pericardial effusion | NA | 3.61 (1.56 – 8.40), p=0.003 |
| LV eccentric | NA | 2.52 (1.19 – 5.34), p=0.02 |
| Caval respiratory variation | NA | 0.28 (0.13 – 0.61), p=0.001 |
| Ischemic heart disease | 3.67 (2.05 – 6.58), p<0.001 | 3.40 (1.75 – 6.58), p<0.001 |
| Chronic kidney disease | 3.81 (1.36 – 10.66), p=0.01 | 5.58 (1.97 – 15.81), p=0.001 |
| Hematological disease | 3.07 (1.54 – 6.10), p=0.001 | 2.21 (0.94 – 5.24), p=0.07 |
| Innate heart defect | 4.13 (1.28 – 13.31), p=0.02 | NA |
| Cancer | 2.18 (1.16 – 4.10), p=0.01 | 2.42 (1.21 – 4.81), p=0.01 |
| PEA surgery | NA | 0.41 (0.18 – 0.92), p=0.03 |

* Scaled to aid presentation. Abbreviations: NA = coverage <70%, NYHA FC = New York Heart Association Functional Class, NT-proBNP = N-terminal prohormone of brain natriuretic peptide, eGFR = estimated glomerular filtration rate, 6MWD = Six-minute walking distance, DLCOc = diffusing capacity of the lungs for carbon monoxide, RHC = right heart catheterization, PAP = pulmonary arterial pressure, PCWP = pulmonary capillary wedge pressure, PVR = pulmonary vascular resistance, TAPSE = tricuspid annular plane systolic excursion, LV = left ventricle, PEA = pulmonary endarterectomy. For the full list of variables considered for an association with survival, please see the supplementary appendix.

**Table S2. List of variables considered for an association with survival.** Variables are **bolded** if p<0.01. The p-value limit is arbitrary, as this study was not designed to test the predictive power of the variables. Variables with perfect separation are indicated by * and NA when there are not enough observations. See also Table 4 in the article. Cox PH summaries are reported as HR (95% CI), p-value, % missing.

| **Variable** | **PAH** | |
| --- | --- | --- |
|  | Diagnosis | 12 months |
| Follow-up related  Date of diagnosis (per year)  Diagnosis before 2015-09-30 | 0.99 (0.94 – 1.05), 0.83, 0%  1.28 (0.83 – 1.99), 0.26, 0% | 0.99 (0.92 – 1.06), 0.79, 0%  1.22 (0.74 – 2.02), 0.44, 0% |
| Demographics  Age at diagnosis  Sex  Smoking: Ever  Smoking: Yes or snuff  Smoking: Start year  Smoking: Pack years  Alcohol use: Weekly units | **1.05 (1.03 – 1.06), <0.001, 0%**  1.32 (0.90 – 1.93), 0.16, 0%  0.79 (0.54 – 1.16), 0.23, 14%  0.58 (0.27 – 1.26), 0.17, 14%  0.97 (0.91 – 1.04), 0.44, 95%  **1.04 (1.02 – 1.07), <0.001, 81%**  0.98 (0.91 – 1.06), 0.59, 70% | **1.05 (1.03 – 1.06), <0.001, 0%**  1.35 (0.89 – 2.07), 0.16, 0%  0.74 (0.48 – 1.13), 0.16, 14%  0.29 (0.09 – 0.92), 0.04, 14%  0.95 (0.87 – 1.04), 0.26, 95%  **1.05 (1.02 – 1.08), <0.001, 80%**  0.97 (0.89 – 1.06), 0.52, 69% |
| PH type  IPAH  APAH connective  APAH congenital heart disease  APAH portal hypertension  HPAH  PVOD or PCH  Drugs or toxins | 0.88 (0.61 – 1.26), 0.48, 0%  **2.15 (1.48 – 3.13), <0.001, 0%**  0.60 (0.35 – 1.02), 0.06, 0%  0.54 (0.13 – 2.20), 0.39, 0%  0.18 (0.03 – 1.32), 0.09, 0%  1.70 (0.86 – 3.35), 0.13, 0%  * | 0.86 (0.58 – 1.28), 0.46, 0%  **2.17 (1.43 – 3.31), <0.001, 0%**  0.66 (0.38 – 1.16), 0.15, 0%  0.66 (0.16 – 2.68), 0.56, 0%  0.22 (0.03 – 1.60), 0.14, 0%  1.61 (0.75 – 3.48), 0.22, 0%  * |
| Clinical characteristics  Hereditary PH  Mutation tested: no mutation  Chest pain  Right heart insufficiency  Weight  Height  BMI  Systolic blood pressure  Diastolic blood pressure  Obesity (BMI≥30) (assume no measurement means no condition)  Heart rate  O2 saturation  Need for additional oxygen  NYHA FC 3 (NYHA FC 1/2 reference)  NYHA FC 4 (NYHA FC 1/2 reference)  Pacemaker | 0.44 (0.14 – 1.39), 0.16, 23%  *  0.72 (0.32 – 1.66), 0.44, 15%  **1.80 (1.19 – 2.73), 0.006, 12%**  1.00 (0.99 – 1.01), 0.49, 29%  0.99 (0.97 – 1.01), 0.41, 29%  0.99 (0.96 – 1.03), 0.60, 38%  0.99 (0.99 – 1.00), 0.22, 15%  0.99 (0.98 – 1.01), 0.38, 15%  0.78 (0.47 – 1.31), 0.35, 0%  1.01 (1.00 – 1.02), 0.19, 33%  **0.93 (0.90 – 0.96), <0.001, 31%**  1.02 (1.00 – 1.04), 0.04, 66%  1.60 (1.02 – 2.51), 0.04, 8%  2.05 (1.12 – 3.75), 0.02, 8%  1.42 (0.45 – 4.47), 0.55, 0% | 0.58 (0.18 – 1.85), 0.36, 24%  *  0.86 (0.27 – 2.74), 0.80, 10%  **2.44 (1.51 – 3.95), <0.001, 7%**  1.00 (0.99 – 1.02), 0.58, 59%  NA  1.02 (0.96 – 1.09), 0.52, 65%  1.00 (0.99 – 1.01), 0.75, 41%  0.99 (0.97 – 1.02), 0.59, 41%  0.86 (0.38 – 1.98), 0.73, 0%  1.04 (1.01 – 1.07), 0.01, 54%  **0.90 (0.84 – 0.96), <0.001, 51%**  0.98 (0.95 – 1.02), 0.34, 73%  **3.69 (2.21 – 6.14), <0.001, 33%**  **33.94 (8.80 – 130.96), <0.001, 33%**  0.96 (0.24 – 3.90), 0.95, 0% |
| Blood test  Albumin  Bilirubin  BNP  NT-proBNP / 1000  Hemoglobin  Creatinine  eGFR  Urate  ASAT  ALAT  TnT  TnI  Prealbumin | 1.00 (0.96 – 1.05), 0.91, 52%  **1.02 (1.01 – 1.04), 0.009, 32%**  1.00 (1.00 – 1.00), 0.01, 85%  **1.09 (1.04 – 1.14), <0.001, 25%**  1.00 (0.99 – 1.00), 0.33, 8%  1.00 (1.00 – 1.00), 0.13, 9%  **0.98 (0.98 – 0.99), <0.001, 9%**  **1.00 (1.00 – 1.00), 0.008, 54%**  1.00 (0.98 – 1.02), 0.96, 44%  0.99 (0.98 – 1.00), 0.28, 18%  **1.05 (1.03 – 1.06), <0.001, 46%**  1.04 (1.01 – 1.07), 0.02, 89%  1.00 (0.99 – 1.01), 0.38, 97% | 0.96 (0.92 – 1.01), 0.11, 69%  **1.05 (1.02 – 1.07), <0.001, 51%**  1.00 (1.00 – 1.00), 0.02, 87%  **1.08 (1.05 – 1.11), <0.001, 40%**  1.00 (0.99 – 1.01), 0.74, 29%  **1.01 (1.01 – 1.01), <0.001, 32%**  **0.97 (0.96 – 0.98), <0.001, 32%**  **1.01 (1.00 – 1.01), <0.001, 60%**  1.00 (0.98 – 1.03), 0.83, 57%  0.99 (0.97 – 1.01), 0.29, 42%  **1.02 (1.01 – 1.03), <0.001, 59%**  1.03 (0.78 – 1.35), 0.84, 92%  NA |
| ECG  Sinus rhythm  Flimmer  Flutter  Pacemaker  Other rhythm  No bundle-branch block  RBBB  LBBB  IVCD  Heart rate  QRS | 1.01 (0.62 – 1.65), 0.98, 14%  1.43 (0.84 – 2.44), 0.19, 14%  1.56 (0.22 – 11.24), 0.66, 14%  0.98 (0.14 – 7.05), 0.98, 14%  NA  0.86 (0.58 – 1.27), 0.44, 14%  1.34 (0.78 – 2.30), 0.29, 14%  2.26 (0.71 – 7.15), 0.17, 14%  0.63 (0.23 – 1.71), 0.36, 14%  1.01 (1.00, 1.02), 0.13, 25%  1.01 (1.00 – 1.02), 0.23, 40% | 0.83 (0.51 – 1.33), 0.44, 7%  **2.45 (1.49 – 4.03), <0.001, 7%**  *  0.68 (0.09 – 4.87), 0.70, 7%  1.51 (0.21 – 10.90), 0.68, 7%  0.75 (0.50 – 1.13), 0.17, 7%  **2.14 (1.23 – 3.74), 0.008, 7%**  0.81 (0.11 – 5.82), 0.83, 7%  1.46 (0.59 – 3.60), 0.42, 7%  **1.03 (1.01 – 1.06), 0.009, 50%**  1.02 (0.99 – 1.04), 0.15, 67% |
| ECHO  CVP  RV EDD  LV EDD  LV EF  LV eccentric (yes/no)  LV EI  E  A  E’  LVOT VTI  RV+RA  RA  TAPSE  RV S’  RV/RA grad  PA width  PVAT  Notch (yes/no)  PI initial diastole  PI end diastole  LA width  Inferior cava  Caval respiratory variation (yes/no)  Pericardium fluid (yes no)  TAPSE / (RV/RA grad + CVP)  TAPSE / RV/RA grad | 1.00 (0.97 – 1.04), 0.98, 62%  1.02 (0.99 – 1.06), 0.21, 56%  1.01 (0.98 – 1.04), 0.50, 10%  1.00 (0.98 – 1.02), 0.80, 13%  1.16 (0.64 – 2.12), 0.63, 44%  1.49 (0.58 – 3.81), 0.40, 80%  1.45 (0.58 – 3.62), 0.42, 51%  **8.88 (3.06 – 25.80), <0.001, 62%**  1.03 (0.93 – 1.14), 0.57, 80%  1.01 (0.89 – 1.16), 0.84, 94%  1.01 (1.00 – 1.02), 0.09, 32%  1.02 (0.99 – 1.05), 0.25, 87%  0.98 (0.94 – 1.02), 0.36, 21%  1.00 (0.88 – 1.15), 0.95, 79%  1.00 (0.99 – 1.01), 0.40, 14%  0.99 (0.92 – 1.06), 0.75, 87%  0.99 (0.96 – 1.02), 0.59, 95%  1.38 (0.12 – 15.45), 0.79, 95%  1.01 (0.94 – 1.09), 0.69, 95%  1.02 (0.98 – 1.07), 0.32, 86%  1.03 (1.00 – 1.05), 0.06, 46%  1.01 (0.97 – 1.05), 0.80, 55%  **0.53 (0.33 – 0.85), 0.009, 45%**  1.00 (0.59 – 1.71), 1.00, 32%  0.89 (0.05 – 16.90), 0.94, 69%  0.67 (0.12 – 3.84), 0.65, 29% | 1.05 (0.99 – 1.13), 0.12, 69%  0.99 (0.94 – 1.03), 0.58, 83%  0.98 (0.95 – 1.01), 0.11, 37%  0.98 (0.95 – 1.01), 0.31, 38%  1.14 (0.68 – 1.91), 0.61, 28%  1.69 (0.66 – 4.29), 0.27, 81%  4.01 (1.34 – 12.00), 0.01, 69%  21.47 (1.20 – 384.72), 0.04, 75%  1.01 (0.83 – 1.22), 0.93, 89%  1.02 (0.87 – 1.20), 0.79, 97%  **1.03 (1.01 – 1.04), <0.001, 45%**  0.98 (0.89 – 1.06), 0.58, 88%  0.92 (0.87 – 0.98), 0.01, 38%  0.96 (0.81 – 1.14), 0.63, 87%  **1.02 (1.01 – 1.04), <0.001, 39%**  0.98 (0.77 – 1.25), 0.87, 93%  1.00 (0.94 – 1.07), 0.97, 94%  1.75 (0.28 – 10.92), 0.55, 90%  1.09 (0.94 – 1.27), 0.26, 97%  0.99 (0.91 – 1.09), 0.86, 90%  1.05 (1.01 – 1.09), 0.03, 64%  1.05 (1.00 – 1.10), 0.06, 63%  **0.37 (0.23 – 0.62), <0.001, 28%**  1.31 (0.63 – 2.73), 0.46, 16%  **0.00 (0.00 – 0.13), 0.005, 73%**  0.03 (0.00 – 0.27), 0.02, 46% |
| Walking test  6MWD/100  Percent of predicted 6MWD  O2 saturation minimum  O2 saturation prior to test  Additional oxygen | **0.72 (0.62 – 0.83), <0.001, 30%**  0.45 (0.17 – 1.16), 0.10, 51%  **0.96 (0.94 – 0.98), <0.001, 33%**  **0.89 (0.85 – 0.93), <0.001, 35%**  1.02 (0.93 – 1.12), 0.61, 73% | **0.57 (0.46 – 0.70), <0.001, 42%**  NA  0.96 (0.93 – 1.00), 0.02, 45%  0.95 (0.89 – 1.02), 0.17, 47%  **1.36 (1.13 – 1.62), <0.001, 76%** |
| Right heart catheterisation  PCWP  PA systolic  PA diastolic  PA mean  LV EDP  RA/CVP  SvO2  CO  CI  CO method: Fick  CO method: Indirect Fick  CO method: Thermodilution  PVR  Shunt (yes/no)  Qp/Qs  R -> L  Vasoreactive | 1.02 (0.97 – 1.06), 0.50, 21%  0.99 (0.98 – 1.00), 0.10, 12%  0.98 (0.96 – 1.00), 0.10, 14%  0.98 (0.96 – 1.00), 0.06, 9%  1.04 (0.95 – 1.13), 0.40, 83%  0.99 (0.97 – 1.02), 0.59, 47%  1.00 (0.97 – 1.03), 0.98, 58%  0.85 (0.73 – 0.99), 0.04, 23%  **0.65 (0.47 – 0.89), 0.008, 34%**  *  0.90 (0.62 – 1.30), 0.57, 87%  0.76 (0.61 – 0.94), 0.01, 54%  1.01 (0.96 – 1.06), 0.64, 20%  0.86 (0.43 – 1.72), 0.67, 30%  0.09 (0.01 – 1.27), 0.07, 96%  0.96 (0.88 – 1.05), 0.35, 98%  0.70 (0.40 – 1.23), 0.22, 44% | 1.01 (0.87–1.17), 0.88, 95%  1.02 (0.97–1.08), 0.47, 94%  1.00 (0.90–1.12), 1.00, 94%  1.04 (0.98–1.11), 0.22, 93%  NA  1.20 (0.97–1.48), 0.09, 96%  0.94 (0.73–1.21), 0.64, 98%  0.71 (0.29–1.70), 0.44, 95%  0.37 (0.07–1.90), 0.23, 95%  NA  NA  0.77 (0.36–1.62), 0.49, 97%  1.08 (0.95–1.23), 0.25, 94%  0.93 (0.42–2.04), 0.86, 30%  NA  NA  0.79 (0.43 – 1.42), 0.43, 43% |
| Spirometry  FEV1  FEV1 %  Z-value  FVC  FVC %  DLCOc  DLCOc < 45 (assume no measurement means not <45)  DLCOc/VA | 1.02 (1.00 – 1.05), 0.08, 42%  1.01 (1.00 – 1.02), 0.31, 42%  1.42 (0.59 – 3.42), 0.43, 91%  0.85 (0.64 – 1.12), 0.25, 43%  1.01 (1.00 – 1.03), 0.14, 43%  **0.97 (0.95 – 0.98), <0.001, 49%**  **2.09 (1.40 – 3.14), <0.001, 0%**  **0.97 (0.96 – 0.98), <0.001, 52%** | 0.35 (0.10 – 1.20), 0.09, 91%  0.97 (0.95 – 1.00), 0.09, 92%  NA  0.39 (0.14 – 1.04), 0.06, 91%  NA  0.99 (0.94 – 1.04), 0.76, 95%  0.94 (0.30 – 2.98), 0.92, 0%  0.98 (0.92 – 1.04), 0.43, 95% |
| Ventilation/perfusion map  Embolism (yes/no) | 0.44 (0.19 – 1.00), 0.05, 38% | 0.32 (0.12 – 0.88), 0.03, 36% |
| Pulmonary CT angiography  PA  Embolism (yes/no) | 0.98 (0.93 – 1.04), 0.56, 79%  0.85 (0.31 – 2.34), 0.75, 43% | *  1.51 (0.60 – 3.83), 0.38, 41% |
| Pulmonary HR CT  Emphysema  Parenchymal disease  PVOD  Normal  PCH  Other | 1.81 (0.93 – 3.53), 0.08, 40%  1.07 (0.59 – 1.93), 0.83, 40%  0.60 (0.15 – 2.45), 0.48, 40%  0.78 (0.50 – 1.21), 0.26, 40%  11.57 (1.51 – 88.44), 0.02, 40%  1.38 (0.88 – 2.16), 0.16, 40% | 2.35 (1.19 – 4.63), 0.01, 39%  0.99 (0.49 – 1.99), 0.97, 39%  0.81 (0.20 – 3.32), 0.77, 39%  0.80 (0.49 – 1.31), 0.37, 39%  NA  0.99 (0.60 – 1.64), 0.97, 39% |
| Pulmonary angiography  No changes  Proximal changes  Distal changes | 0.45 (0.02 – 8.98), 0.60, 97%  NA  NA | NA  NA  NA |
| Spiroergometry  VO2 max  VO2 max %  VE/VCO2 slope | 0.99 (0.78 – 1.25), 0.92, 96%  1.01 (0.95 – 1.07), 0.73, 97%  1.10 (0.95 – 1.27), 0.22, 98% | NA  NA  NA |
| Comorbidities  Atrial fibrillation  Atrial flutter  Cancer  CKD  Deep vein thrombosis  Diabetes type 1  Diabetes type 2  Hematological disease  Innate heart defect (any)  Innate heart defect, ASD  Innate heart defect, corrected  Innate heart defect, Eisenmenger  Innate heart defect, PDA  Innate heart defect, VSD  Innate heart defect, other  Innate heart defect: LR, small, other  Ischemic heart disease  Left heart insufficiency (any)  Left heart insufficiency: diastolic  Left heart insufficiency: systolic  Lung disease (any)  Lung disease: Asthma  Lung disease: COPD  Lung disease: Parenchymal  Lung disease: Other  Lung disease: COPD or emphysema  Lung embolism  Portal hypertension  Systemic sclerosis or CREST  Sleep apnea  Stroke  Thrombophilia  Thyroid disease (any)  Hyperthyroidism  Hypothyroidism  Transient ischemic attack  Treated hypertension  Valvular disease (any)  Valvular disease: AS  Valvular disease: MI  Valvular disease: TI  Valvular disease: other  Other comorbidity | 1.34 (0.87 – 2.07), 0.18, 0%  0.80 (0.25 – 2.52), 0.70, 0%  1.31 (0.76 – 2.25), 0.33, 0%  1.32 (0.61 – 2.83), 0.48, 0%  0.55 (0.20 – 1.48), 0.23, 0%  0.27 (0.04 – 1.94), 0.19, 0%  **2.08 (1.38 – 3.14), <0.001, 0%**  1.04 (0.46 – 2.37), 0.92, 0%  0.59 (0.35 – 1.01), 0.05, 0%  0.55 (0.24 – 1.25), 0.15, 0%  0.46 (0.15 – 1.46), 0.19, 0%  0.24 (0.03 – 1.77), 0.16, 0%  1.23 (0.39 – 3.87), 0.73, 0%  1.00 (0.32 – 3.15), 1.00, 0%  1.07 (0.50 – 2.30), 0.86, 0%  1.11 (0.54 – 2.28), 0.77, 0%  **2.09 (1.33 – 3.26), 0.001, 0%**  1.19 (0.60 – 2.35), 0.61, 0%  1.15 (0.47 – 2.82), 0.76, 0%  1.23 (0.45 – 3.33), 0.68, 0%  1.46 (1.00 – 2.13), 0.05, 0%  0.59 (0.32 – 1.10), 0.10, 0%  1.14 (0.50 – 2.58), 0.76, 0%  1.86 (0.94 – 3.68), 0.07, 0%  1.92 (1.13 – 3.26), 0.02, 0%  1.31 (0.64 – 2.68), 0.46, 0%  0.52 (0.26 – 1.02), 0.06, 0%  0.77 (0.25 – 2.43), 0.66, 0%  **3.30 (2.21 – 4.92), <0.001, 0%**  0.84 (0.43 – 1.66), 0.62, 0%  2.35 (0.95 – 5.78), 0.06, 0%  0.35 (0.05 – 2.50), 0.29, 0%  0.80 (0.48 – 1.32), 0.38, 0%  0.88 (0.28 – 2.77), 0.83, 0%  0.79 (0.46 – 1.36), 0.40, 0%  2.59 (0.95 – 7.08), 0.06, 0%  1.40 (0.98 – 2.00), 0.06, 0%  1.35 (0.84 – 2.16), 0.21, 0%  1.83 (0.58 – 5.78), 0.30, 0%  0.43 (0.06 – 3.11), 0.41, 0%  1.76 (1.02 – 3.02), 0.04, 0%  1.33 (0.19 – 9.57), 0.77, 0%  0.80 (0.54 – 1.19), 0.28, 0% | 1.54 (0.97 – 2.45), 0.07, 0%  1.03 (0.33 – 3.26), 0.96, 0%  1.27 (0.69 – 2.32), 0.44, 0%  1.47 (0.64 – 3.36), 0.36, 0%  0.50 (0.16 – 1.58), 0.24, 0%  0.30 (0.04 – 2.17), 0.23, 0%  **2.22 (1.40 – 3.51), <0.001, 0%**  0.61 (0.19 – 1.92), 0.40, 0%  0.66 (0.38 – 1.15), 0.14, 0%  0.65 (0.28 – 1.48), 0.30, 0%  0.54 (0.17 – 1.72), 0.30, 0%  *  0.92 (0.23 – 3.75), 0.91, 0%  1.19 (0.38 – 3.75), 0.77, 0%  1.33 (0.61 – 2.87), 0.47, 0%  3.31 (0.81 – 13.48), 0.09, 0%  **2.43 (1.50 – 3.94), <0.001, 0%**  1.13 (0.52 – 2.43), 0.76, 0%  1.43 (0.58 – 3.52), 0.44, 0%  0.73 (0.18 – 2.97), 0.66, 0%  1.43 (0.94 – 2.18), 0.09, 0%  0.58 (0.29 – 1.16), 0.12, 0%  1.17 (0.47 – 2.87), 0.74, 0%  2.00 (0.93 – 4.34), 0.08, 0%  1.70 (0.90 – 3.19), 0.10, 0%  1.42 (0.66 – 3.06), 0.37, 0%  0.47 (0.22 – 1.03), 0.06, 0%  0.96 (0.30 – 3.03), 0.94, 0%  **3.46 (2.19 – 5.45), <0.001, 0%**  1.07 (0.54 – 0.84), 0.84, 0%  **3.37 (1.36 – 8.37), 0.009, 0%**  0.40 (0.06 – 2.88), 0.36, 0%  0.84 (0.48 – 1.45), 0.52, 0%  1.09 (0.34 – 3.44), 0.89, 0%  0.79 (0.43 – 1.45), 0.45, 0%  **4.02 (1.46 – 11.08), 0.007, 0%**  1.56 (1.05 – 2.30), 0.03, 0%  1.35 (0.80 – 2.27), 0.26, 0%  2.36 (0.74 – 7.51), 0.15, 0%  0.52 (0.07 – 3.71), 0.51, 0%  1.82 (0.99 – 3.32), 0.05, 0%  1.52 (0.21 – 10.93), 0.68, 0%  0.86 (0.56 – 1.31), 0.47, 0% |
| Derived risk factors  3+ cardiovascular comorbidities (assume not obese with no measurements)  Cardiopulmonary phenotype  Any CTEPH risk factor | 1.51 (0.83 – 2.75), 0.18, 0%  **1.99 (1.18 – 3.35), 0.009, 14%**  0.85 (0.55 – 1.16), 0.24, 0% | **­2.54 (1.31 – 4.93), 0.006, 0%**  0.75 (0.10 – 5.43), 0.78, 14%  0.68 (0.45 – 1.04), 0.08, 0% |

| **Variable** | **CTEPH** | |
| --- | --- | --- |
|  | Diagnosis | 12 months |
| Follow-up related  Date of diagnosis (per year)  Diagnosis before 2015-09-30 | 0.90 (0.81 – 0.99), 0.03, 0%  1.39 (0.68 – 2.83), 0.36, 0% | 0.92 (0.82 – 1.04), 0.18, 0%  1.26 (0.55 – 2.89), 0.59, 0% |
| Demographics  Age at diagnosis  Sex  Smoking: Ever  Smoking: Yes or snuff  Smoking: Start year  Smoking: Pack years  Alcohol use: Weekly units | **1.08 (1.04 – 1.11), <0.001, 0%**  0.84 (0.49 – 1.42), 0.51, 0%  0.59 (0.33 – 1.06), 0.08, 11%  0.54 (0.17 – 1.73), 0.30, 11%  0.92 (0.66 – 1.28), 0.63, 97%  1.00 (0.99 – 1.01), 0.69, 78%  0.89 (0.75 – 1.06), 0.18, 75% | **1.10 (1.05 – 1.14), <0.001, 0%**  0.89 (0.49 – 1.60), 0.69, 0%  0.63 (0.33 – 1.18), 0.15, 10%  0.64 (0.20 – 2.08), 0.46, 11%  0.92 (0.66 – 1.28), 0.63, 96%  1.00 (0.99 – 1.01), 0.70, 78%  0.94 (0.80 – 1.11), 0.45, 75% |
| Clinical characteristics  Hereditary PH  Mutation tested: no mutation  Chest pain  Right heart insufficiency  Weight  Height  BMI  Systolic blood pressure  Diastolic blood pressure  Obesity (BMI≥30) (assume no measurement means no condition)  Heart rate  O2 saturation  Need for additional oxygen  NYHA FC 3 (NYHA FC 1/2 reference)  NYHA FC 4 (NYHA FC 1/2 reference)  Pacemaker | *  1.47 (0.36 – 6.04), 0.59, 0%  2.11 (0.75 – 5.94), 0.16, 15%  **3.60 (1.84 – 7.04), <0.001, 14%**  1.00 (0.98 – 1.02), 0.84, 28%  0.97 (0.93 – 1.00), 0.05, 27%  1.01 (0.95 – 1.08), 0.71, 33%  0.98 (0.97 – 1.00), 0.07, 11%  0.97 (0.94 – 0.99), 0.01, 11%  0.58 (0.26 – 1.29), 0.18, 0%   - 1. (0.99 – 1.03), 0.47, 27%   0.99 (0.97 – 1.00), 0.14, 24%  1.01 (0.99 – 1.03), 0.44, 61%  1.71 (0.78 – 3.77), 0.18, 5%  **6.83 (2.82 – 16.54), <0.001, 5%**  1.33 (0.32 – 5.49), 0.69, 0% | *  1.77 (0.43 – 7.32), 0.43, 0%  3.02 (1.06 – 8.60), 0.04, 12%  **3.27 (1.55 – 6.90), 0.002, 9%**  0.96 (0.93 – 1.00), 0.05, 59%  NA  0.89 (0.78 – 1.02), 0.09, 64%  0.98 (0.95 – 1.00), 0.08, 43%  0.95 (0.91 – 0.99), 0.03, 43%  0.18 (0.02 – 1.28), 0.09, 0%  1.00 (0.97 – 1.03), 0.92, 51%  **0.85 (0.75 – 0.96), 0.007, 51%**  1.41 (1.06 – 1.88), 0.02, 27%  **6.10 (2.68 – 13.87), <0.001, 34%**  5.80 (0.73 – 46.40), 0.10, 34%  1.81 (0.44 – 7.55), 0.41, 0% |
| Blood test  Albumin  Bilirubin  BNP  NT-proBNP / 1000  Hemoglobin  Creatinine  eGFR  Urate  ASAT  ALAT  TnT  TnI  Prealbumin | 0.90 (0.82 – 0.97), 0.01, 57%  1.01 (0.99 – 1.02), 0.24, 40%  1.00 (1.00 – 1.01), 0.10, 85%  **1.14 (1.09 – 1.20), <0.001, 22%**  **0.97 (0.95 – 0.98), <0.001, 6%**  **1.01 (1.00 – 1.02), 0.003, 7%**  **0.97 (0.95 – 0.99), <0.001, 7%**  1.00 (1.00, 1.01), 0.06, 55%  1.01 (0.98 – 1.03), 0.58, 44%  **1.00 (1.00 – 1.01), 0.008, 20%**  1.00 (1.00 – 1.01), 0.83, 48%  1.01 (1.00 – 1.02), 0.05, 89%  NA | 0.95 (0.86 – 1.06), 0.38, 75%  1.01 (0.99 – 1.03), 0.17, 55%  1.01 (1.00 – 1.01), 0.08, 88%  **1.42 (1.23 – 1.63), <0.001, 43%**  **0.97 (0.95 – 0.99), 0.003, 36%**  1.01 (1.00 – 1.02), 0.10, 36%  0.97 (0.96 – 0.99), 0.01, 36%  **1.01 (1.00 – 1.01), 0.002, 66%**  1.01 (0.97 – 1.05), 0.65, 57%  0.97 (0.94 – 1.01), 0.15, 43%  1.01 (1.00 – 1.02), 0.07, 64%  1.03 (0.98 – 1.08), 0.25, 95%  NA |
| EKG  Sinus rhythm  Flimmer  Flutter  Pacemaker  Other rhythm  No bundle-branch block  RBBB  LBBB  IVCD  Heart rate  QRS | **0.31 (0.13 – 0.74), 0.008, 13%**  **4.69 (1.62 – 13.55), 0.004, 13%**  8.72 (1.16 – 65.51), 0.04, 13%  1.13 (0.15 – 8.20), 0.91, 13%  *  0.91 (0.48 – 1.72), 0.77, 13%  1.10 (0.43 – 2.81), 0.84, 13%  *  *  1.02 (1.00 – 1.04), 0.08, 24%  1.01 (0.99 – 1.04), 0.29, 36% | 0.42 (0.18 – 1.00), 0.05, 3%  **4.55 (1.58 – 13.10), 0.005, 3%**  1.11 (0.15 – 8.10), 0.92, 3%  0.99 (0.13 – 7.20), 0.99, 3%  NA  0.77 (0.41 – 1.45), 0.42, 3%  1.07 (0.45 – 2.55), 0.88, 3%  NA  *  1.01 (0.98 – 1.04), 0.57, 47%  **1.04 (1.01 – 1.06), 0.004, 59%** |
| ECHO  CVP  RV EDD  LV EDD  LV EF  LV eccentric (yes/no)  LV EI  E  A  E’  LVOT VTI  RV+RA  RA  TAPSE  RV S’  RV/RA grad  PA width  PVAT  Notch (yes/no)  PI initial diastole  PI end diastole  LA width  Inferior cava  Caval respiratory variation (yes/no)  Pericardium fluid (yes no)  TAPSE / (RV/RA grad + CVP)  TAPSE / RV/RA grad | - 1. (0.97 – 1.07), 0.44, 60%   **1.07 (1.02 – 1.13), 0.007, 61%**  0.99 (0.95 – 1.04), 0.81, 14%  1.01 (0.97 – 1.04), 0.73, 15%  2.19 (0.85 – 5.64), 0.11, 46%  0.56 (0.08 – 4.11), 0.57, 82%  3.79 (0.64 – 22.30), 0.14, 51%  8.04 (0.74 – 87.83), 0.09, 55%  0.90 (0.75 – 1.09), 0.29, 79%  0.84 (0.57 – 1.23), 0.37, 93%  1.00 (0.99 – 1.01), 0.83, 27%  1.01 (0.91 – 1.13), 0.82, 90%  **0.90 (0.84 – 0.97), 0.004, 20%**  0.93 (0.73 – 1.18), 0.56, 80%  1.01 (1.00 – 1.03), 0.09, 15%  1.26 (1.01 – 1.55), 0.04, 93%  1.03 (0.93 – 1.14), 0.56, 93%  *  1.02 (0.95 – 1.08), 0.63, 96%  1.00 (0.93 – 1.07), 0.97, 86%  1.07 (0.99 – 1.15), 0.07, 45%  1.02 (0.96 – 1.09), 0.50, 60%  0.36 (0.17 – 0.79), 0.01, 48%  2.06 (0.79 – 5.38), 0.14, 34%  0.00 (0.00 – 1.25), 0.06, 68%  **0.01 (0.00 – 0.30), 0.008, 30%** | 0.96 (0.81 – 1.13), 0.60, 74%  1.03 (0.94 – 1.13), 0.51, 86%  0.95 (0.88 – 1.02), 0.15, 45%  0.99 (0.94 – 1.04), 0.63, 47%  2.52 (1.19 – 5.34), 0.02, 27%  3.21 (0.12 – 82.95), 0.48, 91%  0.32 (0.01 – 18.04), 0.58, 71%  0.37 (0.01 – 26.91), 0.65, 74%  0.67 (0.39 – 1.14), 0.14, 88%  0.99 (0.80 – 1.21), 0.90, 95%  **1.07 (1.04 – 1.10), <0.001, 48%**  1.11 (0.91 – 1.34), 0.32, 93%  0.93 (0.85 – 1.03), 0.15, 44%  0.68 (0.37 – 1.23), 0.20, 89%  **1.04 (1.02 – 1.06), <0.001, 46%**  NA  NA  *  0.97 (0.79 – 1.20), 0.80, 97%  0.96 (0.67 – 1.37), 0.83, 91%  1.02 (0.91 – 1.13), 0.79, 68%  1.07 (0.97 – 1.19), 0.18, 80%  **0.28 (0.13 – 0.61), 0.001, 29%**  **3.61 (1.56 – 8.40), 0.003, 14%**  0.00 (0.00 – 1.08), 0.05, 78%  **0.00 (0.00 – 0.14), 0.002, 49%** |
| Walking test  6MWD/100  Percent of predicted 6MWD  O2 saturation minimum  O2 saturation prior to test  Additional oxygen | **0.54 (0.41 – 0.71), <0.001, 33%**  **0.10 (0.02 – 0.47), 0.003, 48%**  0.96 (0.91 – 1.02), 0.20, 36%  0.94 (0.87 – 1.02), 0.11, 36%  **1.73 (1.20 – 2.48), 0.003, 69%** | **0.45 (0.30 – 0.67), <0.001, 48%**  NA  **0.89 (0.82 – 0.97), 0.006, 49%**  **0.84 (0.73 – 0.95), 0.008, 52%**  3.21 (0.12 – 82.95), 0.48, 91% |
| Right heart catheterisation  PCWP  PA systolic  PA diastolic  PA mean  LV EDP  RA/CVP  SvO2  CO  CI  CO method: Fick  CO method: Indirect Fick  CO method: Thermodilution  PVR  Shunt (yes/no)  Qp/Qs  R -> L  Vasoreactive | **1.10 (1.06 – 1.15), <0.001, 17%**  1.01 (1.00 – 1.02), 0.19, 10%  **1.04 (1.01 – 1.07), 0.006, 12%**  1.03 (1.00 – 1.06), 0.03, 9%  1.05 (0.89 – 1.23), 0.55, 85%  **1.14 (1.04 – 1.24), 0.005, 43%**  0.95 (0.92 – 1.00), 0.03, 58%  0.84 (0.66 – 1.07), 0.15, 24%  0.62 (0.36 – 1.07), 0.09, 34%  NA  0.52 (0.26 – 1.08), 0.08, 82%  0.95 (0.72 – 1.25), 0.69, 58%  1.07 (1.00 – 1.14), 0.04, 18%  2.31 (0.31 – 17.09), 0.41, 32%  NA  NA  0.95 (0.22 – 4.14), 0.94, 77% | 1.30 (0.83 – 2.04), 0.25, 93%  1.04 (0.93 – 1.16), 0.52, 88%  1.05 (0.88 – 1.27), 0.58, 88%  1.04 (0.90 – 1.20), 0.59, 89%  *  1.05 (0.78 – 1.40), 0.75, 94%  0.94 (0.79 – 1.11), 0.45, 95%  0.66 (0.15 – 2.97), 0.59, 93%  0.49 (0.01 – 29.26), 0.73, 94%  NA  *  NA  0.96 (0.75 – 1.23), 0.74, 94%  2.81 (0.38 – 21.05), 0.31, 32%  NA  NA  0.66 (0.08 – 5.18), 0.69, 79% |
| Spirometry  FEV1  FEV1 %  Z-value  FVC  FVC %  DLCOc  DLCOc < 45 (assume no measurement means not <45)  DLCOc/VA | **0.39 (0.23 – 0.66), <0.001, 41%**  0.98 (0.96 – 1.00), 0.03, 44%  1.36 (0.57 – 3.26), 0.49, 90%  **1.05 (1.02 – 1.09), 0.004, 42%**  0.98 (0.96 – 1.00), 0.07, 45%  0.99 (0.96 – 1.02), 0.45, 54%  1.96 (0.48 – 8.06), 0.35, 0%  0.99 (0.96 – 1.02), 0.51, 54% | NA  NA  NA  NA  NA  NA  NA  NA |
| Ventilation/perfusion map  Embolism (yes/no) | * | * |
| Pulmonary CT angiography  PA  Embolism (yes/no) | 1.05 (0.95 – 1.17), 0.33, 61%  0.71 (0.27 – 1.85), 0.48, 24% | 1.89 (0.53 – 6.74), 0.33, 94%  0.94 (0.35 – 2.50), 0.90, 18% |
| Pulmonary HR CT  Emphysema  Parenchymal disease  PVOD  Normal  PCH  Other | 1.19 (0.25 – 5.72), 0.83, 83%  2.96 (0.61 – 14.34), 0.18, 83%  NA  0.32 (0.06 – 1.60), 0.17, 83%  NA  1.88 (0.57 – 6.21), 0.30, 83% | 1.71 (0.44 – 6.65), 0.44, 81%  2.74 (0.58 – 12.98), 0.21, 81%  NA  0.33 (0.07 – 1.62), 0.17, 81%  NA  1.86 (0.58 – 5.91), 0.30, 81% |
| Pulmonary angiography  No changes  Proximal changes  Distal changes | NA  0.61 (0.21 – 1.81), 0.37, 64%  1.70 (0.52 – 5.55), 0.38, 64% | NA  0.69 (0.22 – 2.20), 0.53, 55%  1.84 (0.55 – 6.14), 0.32, 55% |
| Spiroergometry  VO2 max  VO2 max %  VE/VCO2 slope | NA  NA  NA | NA  NA  NA |
| Comorbidities  Atrial fibrillation  Atrial flutter  Cancer  CKD  Deep vein thrombosis  Diabetes type 1  Diabetes type 2  Hematological disease  Innate heart defect (any)  Innate heart defect, ASD  Innate heart defect, corrected  Innate heart defect, Eisenmenger  Innate heart defect, PDA  Innate heart defect, VSD  Innate heart defect, other  Innate heart defect: LR, small, other  Ischemic heart disease  Left heart insufficiency (any)  Left heart insufficiency: diastolic  Left heart insufficiency: systolic  Lung disease (any)  Lung disease: Asthma  Lung disease: COPD  Lung disease: Parenchymal  Lung disease: Other  Lung disease: COPD or emphysema  Lung embolism  Portal hypertension  Systemic sclerosis or CREST  Sleep apnea  Stroke  Thrombophilia  Thyroid disease (any)  Hyperthyroidism  Hypothyroidism  Transient ischemic attack  Treated hypertension  Valvular disease (any)  Valvular disease: AS  Valvular disease: MI  Valvular disease: TI  Valvular disease: other  Other comorbidity | 2.01 (0.85 – 4.74), 0.11, 0%  4.59 (1.11 – 19.01), 0.04, 0%  2.18 (1.16 – 4.10), 0.01, 0%  3.81 (1.36 – 10.66), 0.01, 0%  0.62 (0.33 – 1.18), 0.14, 0%  *  1.34 (0.60 – 2.99), 0.47, 0%  **3.07 (1.54 – 6.10), 0.001, 0%**  4.13 (1.28 – 13.31), 0.02, 0%  **22.03 (5.02 – 96.59), <0.001, 0%**  NA  NA  NA  2.13 (0.29 – 15.49), 0.46, 0%  1.53 (0.21 – 11.07), 0.68, 0%  1.53 (0.21 – 11.07), 0.68, 0%  **3.67 (2.05 – 6.58), <0.001, 0%**  *  *  *  1.49 (0.86 – 2.59), 0.16, 0%  1.42 (0.77 – 2.61), 0.26, 0%  1.48 (0.63 – 3.47), 0.37, 0%  6.26 (1.50 – 26.06), 0.01, 0%  1.78 (0.64 – 4.93), 0.27, 0%  1.56 (0.70 – 3.46), 0.27, 0%  0.76 (0.32 – 1.78), 0.52, 0%  NA  NA  0.63 (0.20 – 2.03), 0.44, 0%  1.04 (0.37 – 2.88), 0.95, 0%  1.20 (0.51 – 2.81), 0.67, 0%  0.75 (0.27 – 2.07), 0.57, 0%  *  0.76 (0.27 – 2.10), 0.60, 0%  2.07 (0.50 – 8.63), 0.32, 0%  1.27 (0.74 – 2.16), 0.38, 0%  2.21 (1.00 – 4.90), 0.05, 0%  4.11 (1.28 – 13.24), 0.02, 0%  7.05 (0.95 – 52.42), 0.06, 0%  0.81 (0.20 – 3.31), 0.76, 0%  **36.10 (4.22 – 308.98), 0.001, 0%**  1.24 (0.67 – 2.28), 0.49, 0% | 1.82 (0.65 – 5.16), 0.26, 0%  6.20 (1.48 – 25.93), 0.01, 0%  2.42 (1.21 – 4.81), 0.01, 0%  **5.58 (1.97 – 15.81), 0.001, 0%**  0.71 (0.36 – 1.41), 0.33, 0%  *  1.57 (0.66 – 3.75), 0.31, 0%  2.21 (0.94 – 5.24), 0.07, 0%  3.52 (0.85 – 14.65), 0.08, 0%  **27.91 (3.36 – 231.84), 0.002, 0%**  NA  NA  NA  2.42 (0.33 – 17.73), 0.38, 0%  1.84 (0.25 – 13.46), 0.55, 0%  1.84 (0.25 – 13.45), 0.55, 0%  **3.40 (1.75 – 6.58), <0.001, 0%**  *  *  *  1.30 (0.70 – 2.42), 0.41, 0%  1.34 (0.68 – 2.66), 0.40, 0%  1.16 (0.41 – 3.25), 0.78, 0%  **9.59 (2.27 – 40.51), 0.002, 0%**  1.60 (0.49 – 5.19), 0.43, 0%  1.32 (0.52 – 3.36), 0.56, 0%  1.36 (0.42 – 4.41), 0.61, 0%  NA  NA  0.81 (0.25 – 2.63), 0.73, 0%  1.21 (0.43 – 3.38), 0.72, 0%  0.94 (0.34 – 2.62), 0.90, 0%  0.96 (0.34 – 2.68), 0.93, 0%  *  0.96 (0.34 – 2.70), 0.94, 0%  2.36 (0.56 – 9.91), 0.24, 0%  1.34 (0.74 – 2.41), 0.33, 0%  1.92 (0.76 – 4.89), 0.17, 0%  **5.36 (1.64 – 17.44), 0.005, 0%**  *  0.96 (0.23 – 3.99), 0.96, 0%  5.00 (0.68 – 36.92), 0.11,0%  1.24 (0.63 – 2.47), 0.53, 0% |
| Interventions  Elective BPA  Elective PEA | NA  NA | 1.31 (0.39 – 4.44), 0.66, 0%  0.41 (0.18 – 0.92), 0.03, 0% |
| Derived risk factors  3+ cardiovascular comorbidities (assume not obese with no measurements)  Cardiopulmonary phenotype  Any CTEPH risk factor | 1.81 (0.77 – 4.25), 0.17, 0%  2.04 (0.28 – 14.89), 0.48, 11%  0.63 (0.25, 1.59), 0.33, 0% | 1.89 (0.67 – 5.29), 0.23, 0%    NA  0.94 (0.29 – 3.08), 0.92, 0% |
